# Secondhand Smoke Induces Liver Steatosis through Deregulation of Genes Involved in Hepatic Lipid Metabolism

**DOI:** 10.3390/ijms21041296

**Published:** 2020-02-14

**Authors:** Stella Tommasi, Jae-In Yoon, Ahmad Besaratinia

**Affiliations:** 1 Department of Preventive Medicine, USC Keck School of Medicine, University of Southern California, Los Angeles, CA 90033, USA; besarati@med.usc.edu; 2 Department of Cancer Biology, Beckman Research Institute of the City of Hope, Duarte, CA 91010, USA; jiyoon@coh.org

**Keywords:** gene regulation, non-alcoholic fatty liver disease (NAFLD), mouse model, steatosis, tobacco smoke, transcriptome

## Abstract

We investigated the role of secondhand smoke (SHS) exposure, independently of diet, in the development of chronic liver disease. Standard diet-fed mice were exposed to SHS (5 h/day, 5 days/week for 4 months). Genome-wide gene expression analysis, together with molecular pathways and gene network analyses, and histological examination for lipid accumulation, inflammation, fibrosis, and glycogen deposition were performed on the liver of SHS-exposed mice and controls, upon termination of exposure and after one-month recovery in clean air. Aberrantly expressed transcripts were found in the liver of SHS-exposed mice both pre- and post-recovery in clean air (*n* = 473 vs. 222). The persistent deregulated transcripts (*n* = 210) predominantly affected genes and functional networks involved in lipid metabolism as well as in the regulation of the endoplasmic reticulum where manufacturing of lipids occurs. Significant hepatic fat accumulation (steatosis) was observed in the SHS-exposed mice, which progressively increased as the animals underwent recovery in clean air. Moderate increases in lobular inflammation infiltrates and collagen deposition as well as loss of glycogen were also detectable in the liver of SHS-exposed mice. A more pronounced phenotype, manifested as a disrupted cord-like architecture with foci of necrosis, apoptosis, inflammation, and macrovesicular steatosis, was observed in the liver of SHS-exposed mice post-recovery. The progressive accumulation of hepatic fat and other adverse histological changes in the SHS-exposed mice are highly consistent with the perturbation of key lipid genes and associated pathways in the corresponding animals. Our data support a role for SHS in the genesis and progression of metabolic liver disease through deregulation of genes and molecular pathways and functional networks involved in lipid homeostasis.

## 1. Introduction

Non-alcoholic fatty liver disease (NAFLD) is one of the most prevalent forms of chronic liver disorders worldwide [1,2]. The incidence of NAFLD is rising in many parts of the world, especially in developed countries [3]. In the United States alone, between 30% and 40% of the adult population is affected with NAFLD [3]. Among children and adolescents, NAFLD is currently the primary form of liver disease; it is estimated that nearly 10% of the US population aged between 2 and 19 has NAFLD [4]. NAFLD has been associated with insulin resistance and metabolic syndrome—a cluster of conditions consisting of high blood sugar, excess body fat around the waist, and abnormal cholesterol or triglyceride levels—that are key determinants of cardiovascular disease and type 2 diabetes mellitus [1]. Abdominal obesity is a common feature of patients with NAFLD; in obese individuals, the prevalence of NAFLD can exceed 95% [5]. As the epidemic of obesity continues to grow worldwide, so does the prevalence of NAFLD [5].

NAFLD is characterized by the accumulation of fat droplets within the liver cells, a condition known as hepatic ‘*steatosis*’ [6]. Retention of lipids within the cells reflects an impairment of the normal process of synthesis and elimination of fat, primarily triglycerides [6]. Buildup of excess lipids within the cells manifests as the accumulation of vesicles that can displace the nucleus, disrupt cell constituents and, in severe cases, lead to cell rupture/burst [7]. Non-diagnosed and untreated NAFLD can progress from benign steatosis and fatty liver to more permanent and severe liver injury, including non-alcoholic steatohepatitis (NASH) with inflammation and variable fibrosis, cirrhosis, and eventually hepatocellular carcinoma [8].

Although distinct risk factors for NAFLD have been identified, the exact cause(s) of this disease and the underlying mechanisms of its initiation and progression remain unknown. Accumulating evidence shows that exposure to environmental toxicants, including secondhand smoke (SHS), contributes to the development of NAFLD by promoting mitochondrial dysfunction and oxidative stress within the hepatocytes [9,10,11,12]. Several studies have shown that cigarette smoking is an independent risk factor for the onset of NAFLD [13,14,15,16], and significantly associated with increased intrahepatic fat [17] and advanced liver fibrosis [18,19]. Survey based reports have also shown an association between exposure to SHS and development of NAFLD in children [20,21] and in never-smoking women [22]. A more recent systematic review and meta-analysis established that SHS increases the risk of NAFLD approximately 1.38 times [23]. Animal studies have also provided support for a potential role of SHS in the genesis and progression of NAFLD [24,25,26]. Yuan et al. [26] have shown that subchronic exposure of mice to SHS stimulates hepatic fatty acids synthesis by modulating two key regulators of lipid metabolism, including AMP-activated protein kinase (AMPK) and sterol regulatory element binding protein-1c (SREBP-1c). The SHS-exposed mice in the Yuan et al. study developed hepatic steatosis, which—as the authors inferred—would lead to NAFLD development [26]. De la Monte et al. [25] have demonstrated that A/J mice exposed to SHS exhibit progressive liver injury and steatohepatitis, with impairments in hepatic insulin and insulin-like growth factor (IGF) signaling. Azzalini et al. [24] have shown that nose-only exposure of Zucker ‘*obese*’ rats to cigarette smoke, mimicking SHS exposure, worsens the histological severity of NAFLD, increases the burden of oxidative stress, and induces hepatocellular apoptosis [24,27].

Although informative, most animal studies investigating the role of SHS exposure in the development of NAFLD have been conducted in rodents fed with high-fat diets that contain cholate, a known cause of liver inflammation and dysfunction [28]. As a result, the role of SHS exposure, independently of diet, in the genesis of NAFLD is not delineated. In addition, the above studies are limited in scope, as they have focused on the analysis of ‘*select*’ target genes. Therefore, a comprehensive study interrogating the whole transcriptome is needed to establish the global effects of SHS on the regulation of genes that govern NAFLD development. Towards this end, we have constructed the whole hepatic transcriptome in relation to liver histology in ‘standard diet-fed’ mice subchronically exposed to SHS according to our published protocol [29,30,31,32]. More specifically, we have investigated the relationship between global regulation of genes and molecular pathways and gene networks and histological changes indicative of liver injury and hepatic steatosis (i.e., lipid accumulation, inflammation, fibrosis, and glycogen deposition) in the SHS-exposed mice both upon cessation of exposure and after one-month recovery in clean air.

## 2. Results

### 2.1. Genome-Wide Gene Expression Changes in the Liver of Secondhand Smoke (SHS)-Exposed Mice

**Figure 1:**
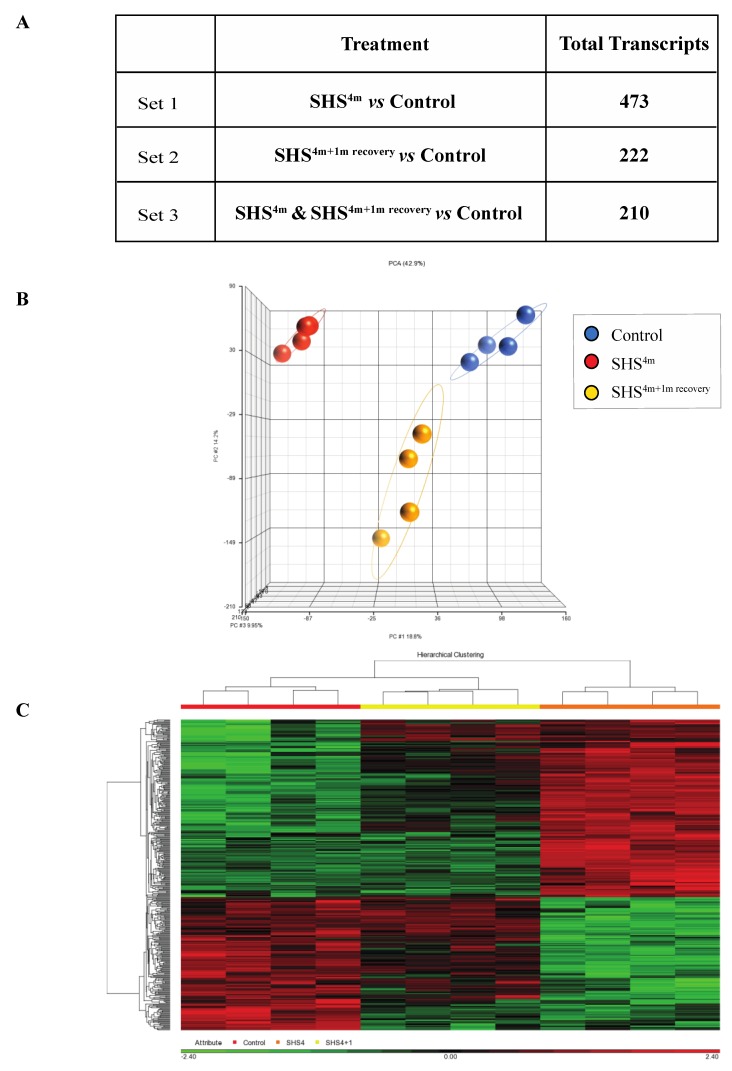
Global gene expression profiling in secondhand smoke (SHS)-exposed mice. (**A**) Differentially expressed transcripts identified in various contrast groups as compared to controls. (**B**) Principal component analysis (PCA) and (**C**) hierarchical clustering analysis by Partek^®^ GS^®^ confirmed clustering of the datasets from mice belonging to the same experimental or control group.

As shown in Figure 1A, subchronic exposure of mice to SHS elicited a significant transcriptomic response, as reflected by the large number of aberrantly expressed transcripts in the SHS-exposed *versus* control mice. More specifically, there were 473 aberrant transcripts in the SHS-exposed mice relative to age-matched controls (Figure 1A; Table S1). One-month recovery in clean air resulted in slight attenuation of the transcriptional changes in the SHS-exposed mice, although the number of aberrantly expressed transcripts remained considerably high in the exposed mice undergone recovery (i.e., 222 transcripts). There were 210 overlapping aberrant transcripts in the SHS-exposed mice pre- and post-recovery.

Principal component analysis (PCA) and hierarchical clustering analysis in Partek GS^®^ showed clustering of the datasets from mice belonging to the same experimental or control groups, which confirms a uniform gene expression pattern within each experimental/control group (Figure 1B,C). Compiled lists of differentially expressed transcripts in experimental groups relative to controls are shown in Table S1. The lists identify both common and unique deregulated genes in the SHS-exposed mice before and after one-month recovery. Overall, there was a high degree of overlap between differentially expressed genes in the SHS-exposed mice before and after recovery in clean air (Figure 1A; Table S1).

### 2.2. Modulation of Functional Networks and Biological Pathways in SHS-Exposed Mice

**Figure 2:**
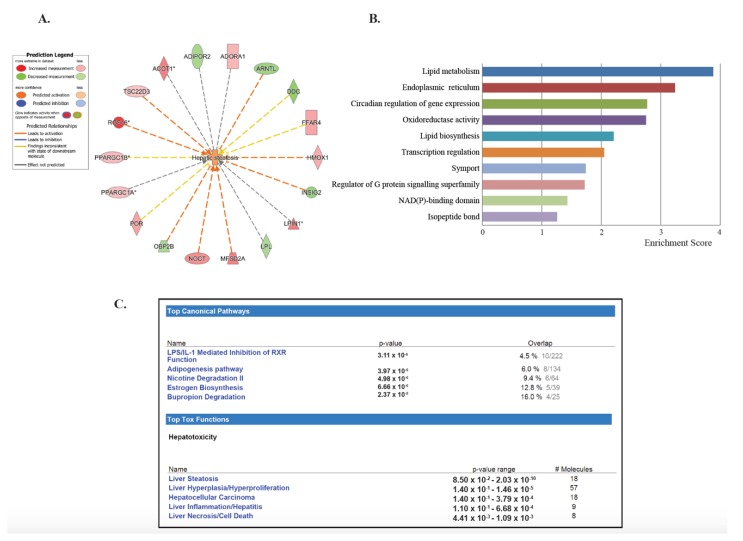
Gene-set enrichment analysis of deregulated genes in SHS-exposed mice. We performed gene ontology analysis on the 153 unique genes identified in SHS-exposed mice, before and after recovery, relative to controls. (**A**) Eighteen genes are specifically implicated in hepatic steatosis. Red and green nodes represent up-regulated and down-regulated genes, respectively. (**B**) The Functional Clustering Analysis tool in DAVID was used to group together redundant annotations. The top ten categories identified by DAVID, with a group enrichment score between 1.26 and 3.88 (*x*-axis), are listed on the *y*-axis. (**C**) The top canonical pathways and hepatotoxic functions were displayed along with the significance values and number of associated molecules and included, among others, liver steatosis, inflammation and necrosis.

To investigate the lasting effects of SHS, we selected the dataset generated by comparing both the SHS^4m^ and SHS^4m+1m recovery^ groups vs. controls (Set 3; Figure 1A). Of the 210 common transcripts, 201 mapped to known IDs, for a total of 153 unique genes (Table 1). Of the 153 differentially expressed genes (DEGs), 63 (>41%) are known to participate in lipid homeostasis, specifically uptake, synthesis, and accumulation of lipids, as well as fatty acids oxidation and secretion (Table 1). Eighteen of these 63 genes (>28%) are specifically involved in liver steatosis (Figure 2A). To characterize the gene networks and functional pathways associated with the 153 unique genes, we performed gene ontology and functional network analyses, using a combination of Database for Annotation, Visualization and Integrated Discovery (DAVID) and Ingenuity*^®^* Pathway Analysis (IPA^®^). Applying the DAVID annotation clustering analysis tool, we discovered twenty-eight relevant biological clusters. The top functional category with the highest enrichment score consisted of gene sets involved in lipid metabolism (Figure 2B). Based on DAVID analysis, we also detected deregulated genes that are involved in oxidoreductase reactions (Figure 2B). The latter is consistent with SHS being a well-known inducer of reactive oxygen species (ROS) and oxidative stress [33,34]. Other highly enriched categories included genes implicated in endoplasmic reticulum function, circadian regulation of gene expression, lipid biosynthesis, and transcription regulation (Figure 2B).

IPA^®^ analysis of the 153 unique DEGs showed disruption of similar gene networks and functional pathways in the SHS-exposed mice, both pre- and post-recovery. As shown in Figure S1, the top impacted networks comprised of genes involved in lipid metabolism and biosynthesis. Other affected gene networks included molecules implicated in behavior and nervous system development and function, cell death and survival, drug metabolism and small molecule biochemistry (Figure S1). The top canonical pathways impacted in the SHS-exposed mice, both pre- and post-recovery, included the lipopolysaccharide (LPS)/IL-1 mediated inhibition of the Retinoid X Receptor (RXR) function (*p* = 3.11 × 10^−6^), the adipogenesis pathway (*p* = 3.97 × 10^−6^), and the nicotine degradation II pathway (*p* = 4.98 × 10^−6^) (Figure 2C). Members of these pathways are known to participate in the negative acute phase response (APR), which down-regulates hepatic genes with crucial physiological roles, in response to liver injury, infection, and/or inflammation. Reduction of key molecules within these pathways ultimately leads to impaired metabolism, transport and/or biosynthesis of lipid, cholesterol, bile acid, and xenobiotics [35].

**Figure 3:**
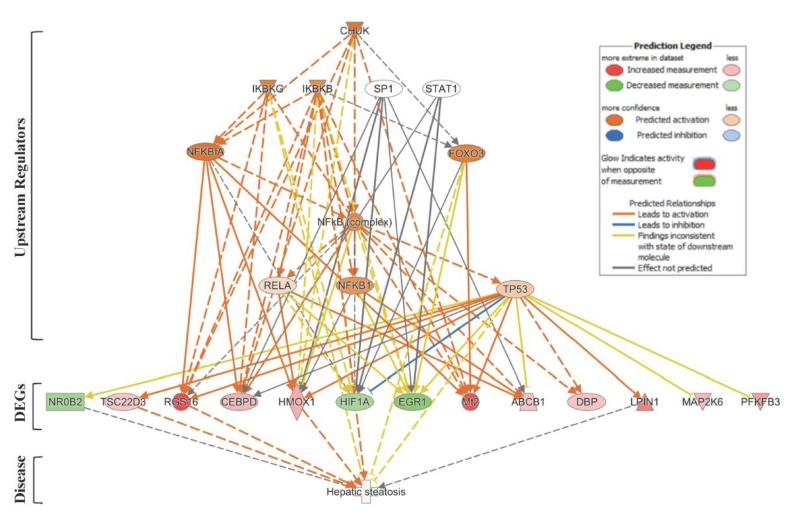
Upstream Regulator Analysis of lipid-specific genes in SHS-exposed mice. We used IPA^®^ Upstream Regulator Analysis to identify the upstream regulators that are likely to account for the aberrant expression of the 63 lipid-specific genes in SHS-exposed mice. Using IPA^®^ prediction analysis, we found that 25 out of the 63 lipid-specific genes are likely to be modulated by a complex network of eleven upstream regulators. For brevity, only gene targets regulated by TP53 are shown. Red molecule, up-regulation; green molecule, down-regulation. Solid and dotted lines indicate a direct or indirect relationship, respectively, between the upstream regulator and its target.

Next, we used the IPA^®^ Upstream Regulator Analysis tool to identify the upstream regulators that are likely to account for the aberrant expression of the 63 lipid-specific genes. Based on IPA^®^ prediction analysis, we identified the Conserved Helix-Loop-Helix Ubiquitous Kinase (CHUK) as the top master regulator with an activation *z*-score of 2.376. CHUK is a member of the serine/threonine protein kinase family, and plays an essential role in the NF-κB signaling pathway. The NF-κB pathway is activated by multiple stimuli, including DNA damage [36] and is involved in inflammation, fibrosis and hepatocarcinogenesis [37]. Our prediction analysis shows that CHUK modulates a complex network of upstream regulators (IKBKG, IKBKB, SP1, STAT1, NFKBIA, FOXO3, NF-κB complex, RELA, NFKB1 and TP53), which may work together to elicit the transcriptional changes observed in a subset of lipid-specific genes (25 out of 63 genes). Thirteen of these 25 target genes are presumably under control of the *TP53* gene (Figure 3). Four of the TP53-regulated genes, i.e., *Rgs16*, *Lpin1*, *Hmox1*, and *Tsc22d3*, are known to be involved in liver steatosis (Figure 2A).

### 2.3. Initiation and Progression of Liver Steatosis in SHS-Exposed Mice

**Figure 4:**
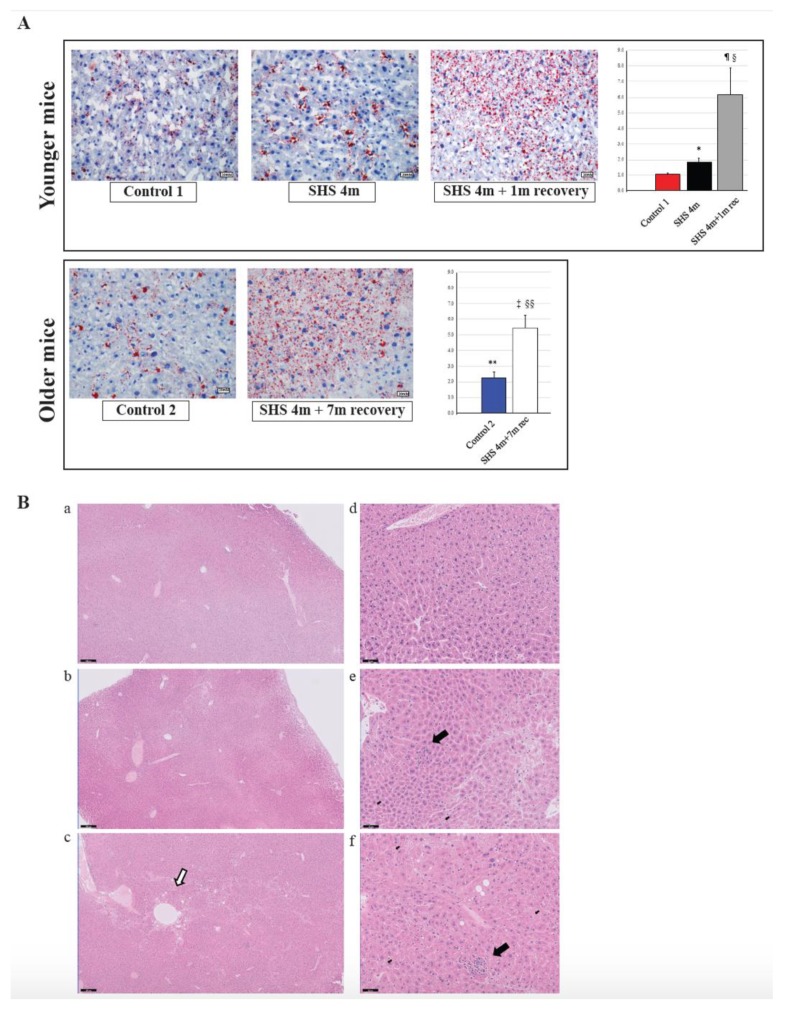
Evaluation of fat accumulation and liver injury in SHS-exposed mice. (**A**) Liver sections from SHS-exposed and control mice were stained with Oil Red O to detect neutral lipid accumulation (red droplets) within the hepatocytes. Microphotographs are shown at 400× original magnifications. Quantification of lipid droplets (graphs on the right) was performed on Oil Red O (ORO)-stained slides (*n* = 4–8) at 400X magnification by measuring the area occupied by red pixels in ImageJ software (https://imagej.nih.gov/ij/). * SHS^4m^ vs. Control 1: *p* = 0.000334; ^¶^ SHS^4m+1m recovery^ vs. Control 1: *p* = 0.000276; ^§^ SHS^4m+1m recovery^ vs. SHS^4m^: *p* = 0.000017; ^‡^ SHS^4m+7m recovery^ vs. Control 2: *p* = 0.000114; ^§§^ SHS^4m+7m recovery^ vs. SHS^4m^: *p* < 0.00001; ** Control 2 vs. Control 1: *p* = 0.000508. (**B**) Paraffin-embedded liver sections from experimental and control mice were stained with hematoxylin and eosin (H&E) to examine cell morphology and detect potential manifestations of liver injury. Representative microphotographs are shown at low (scale bar, 200 μM) and high magnifications (scale bar, 50 μM). Top panels (**a**,**d**), control mice; middle panels (**b**,**e**), SHS^4m^ mice; lower panels (**c**,**f**); SHS^4m+1m recovery^ mice. Small foci of inflammatory infiltrates and areas of necrosis were observed in the liver of SHS^4m^ and SHS^4m+1m recovery^ mice (panels **e**,**f**). SHS^4m+1m recovery^ mice exhibited a disrupted cord-like architecture and a great variability in the size and nuclear morphology of hepatocytes (panel **f**). The white arrow shows an area of pronounced liver steatosis. Big black arrows, foci of inflammation; small black arrows, apoptotic cells.

To investigate whether in vivo exposure of mice to SHS causes anomalies in the lipogenic pathways and induces hepatic steatosis, we performed Oil Red O (ORO) staining on liver sections from SHS-exposed mice in comparison to controls. As illustrated in Figure 4A (upper panels), there was a significant increase in fat deposition (steatosis) within the liver cells of SHS-exposed mice, immediately after treatment, as compared to age-matched controls (*p* = 0.000334). The extent of liver steatosis in the SHS-exposed mice was significantly enhanced after one-month recovery in clean air (SHS^4m+1m recovery^ vs. SHS^4m^; *p* = 0.000017 and SHS^4m+1m recovery^ vs. Control 1; *p* = 0.000276). While reaffirming the steatogenic properties of tobacco smoke on hepatocytes [26,38], these findings show that SHS-induced liver steatosis in vivo is likely to progress and become pervasive. To further determine whether the SHS-induced liver steatosis persisted and/or intensified over prolonged periods of time, we measured fat accumulation in the liver of a subgroup of SHS-exposed mice undergone seven-month recovery in clean air (SHS^4m+7m recovery^). As shown in Figure 4A (lower panels), extended recovery in clean air resulted in progression of the induced liver steatosis in the SHS-exposed mice (SHS^4m+7m recovery^ *vs* SHS^4m^; *p* < 0.00001). The SHS-exposed mice undergone seven-month recovery also showed significantly higher levels of hepatic fat accumulation than age-matched control mice (SHS^4m+7m recovery^ vs. Control 2; *p* = 0.000114).

Ogrodnik et al. [39] have recently shown that cellular senescence drives age-dependent hepatic fat accumulation and steatosis through induction of mitochondrial dysfunction, which, in turn, reduces fat metabolism. Consistent with the findings of that study, we observed higher levels of fat accumulation in the liver of control older mice than younger mice (Control 2 vs. Control 1; *p* = 0.000508). Altogether our data indicate that in vivo exposure of mice to SHS not only initiates liver steatosis but also exacerbates age-dependent progression of hepatic fat deposition.

### 2.4. Histopathological Evaluation of Liver Injury in SHS-Exposed Mice

**Figure 5:**
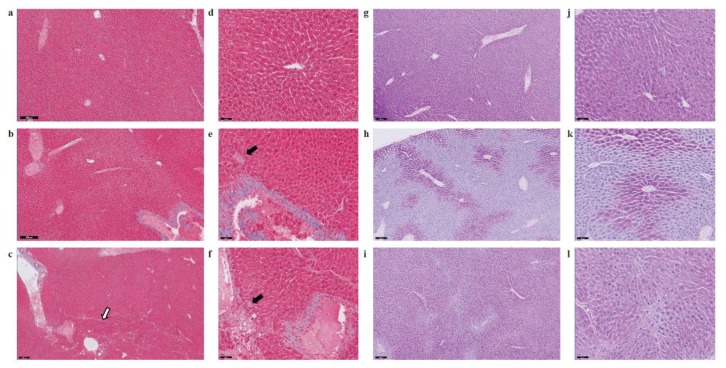
Evaluation of liver fibrosis and glycogen deposition in SHS-exposed mice. Paraffin-embedded liver sections from experimental and control mice were stained with Masson’s trichrome (**a**–**f**) and Periodic Acid-Schiff (PAS) stain (**g**–**l**) to evaluate fibrosis and glycogen deposition, respectively. Representative microphotographs are shown at low (scale bar, 200 μM) and high magnifications (scale bar, 50 μM). Panels **a**, **d**, **g**, **j**, control mice; panels **b**, **e**, **h**, **k**, SHS^4m^ mice; panels **c**, **f**, **i**, **l**, SHS^4m+1m recovery^ mice. Areas of mild liver fibrosis (blue areas) are increasingly observed in the experimental mice. The white arrow shows an area of pronounced liver steatosis. Black arrows indicated parenchymal invasion of collagen fibers. Prominent loss of glycogen was observed in the liver of SHS^4m^ mice, while SHS^4m+1m recovery^ mice and controls show intense and extensive PAS-positive staining.

Several deregulated genes detected in our dataset (*ACOT1*, *ADIPOR2*, *ADORA1*, *EGR1*, *HMOX1*, *IL6R*, *LPIN1*, *NROB2*, and *POR*) are known to play a crucial role in liver inflammation (Table 1). Three genes in particular, ACOT1, ADIPOR2 and ADORA1, are associated with nonalcoholic steatohepatitis [40,41]. Deregulation of IL6, a potent pleiotropic cytokine, and the hepatic IL6 receptor (IL6R) are important contributors to the immune response and acute inflammation in vivo. To further investigate whether exposure to SHS predisposes mice to other forms of liver injury, including inflammation and/or fibrosis, paraffin-embedded liver sections from experimental and control mice were stained with H&E, Masson’s trichrome and Periodic Acid-Schiff (PAS) stain. As shown in Figure 4B and Figure 5, we observed a mild increase in lobular inflammation infiltrates and collagen deposition (blue areas) in the liver of SHS^4m^ and SHS^4m+1m recovery^ mice, as compared to control mice. Consistent with previous findings [25], a more pronounced phenotype manifested in the liver of mice post-recovery. Liver sections from SHS^4m+1m recovery^ mice showed a disrupted cord-like architecture with foci of necrosis, apoptosis, inflammation and macrovesicular steatosis (Figure 4B, panels c,f). A great variability in the size and nuclear morphology of hepatocytes was also observed in these mice (Figure 4B, panel f). Our data show that exposure to SHS is likely to induce early signs of inflammation and fibrosis, with effects that persist even after termination of exposure. Furthermore, the results obtained by histopathological examination are in good agreement with the gene expression data.

A recent study has shown that lack of liver glycogen causes hepatic insulin resistance and steatosis in mice [42]. To examine whether exposure to SHS affects glycogen metabolism, we also performed PAS staining on liver sections from SHS-exposed mice and controls, before and after recovery. As shown in Figure 5, liver tissues from SHS^4m^ mice displayed prominent loss of glycogen (*h*, *k*), while hepatocytes of SHS^4m+1m recovery^ mice and controls showed intense and extensive PAS-positive staining (i, *l*), indicative of glycogen accumulation. The divergent patterns of glycogen loss/buildup in the liver of SHS-exposed vs. control mice are consistent with body weight gains of the corresponding animals (Figure S2). Whilst mice in the control group gained body weight steadily throughout the sham-exposure and subsequent recovery, the mice in the experimental group showed a nearly flat pattern of body weight gain during the four-month SHS exposure. The SHS-exposed mice, however, started to re-gain weight immediately after the termination of exposure (Figure S2) [29]. In confirmation, we observed up-regulation of glycogen synthase 2 (Gys2) in the SHS^4+1m recovery^ mice relative to controls (Table S1), indicating that synthesis of glycogen is resumed following recovery in clean air.

### 2.5. Validation of Genome-Wide Gene Expression Data by Reverse Transcription-Quantitative PCR (RT-qPCR)

**Figure 6:**
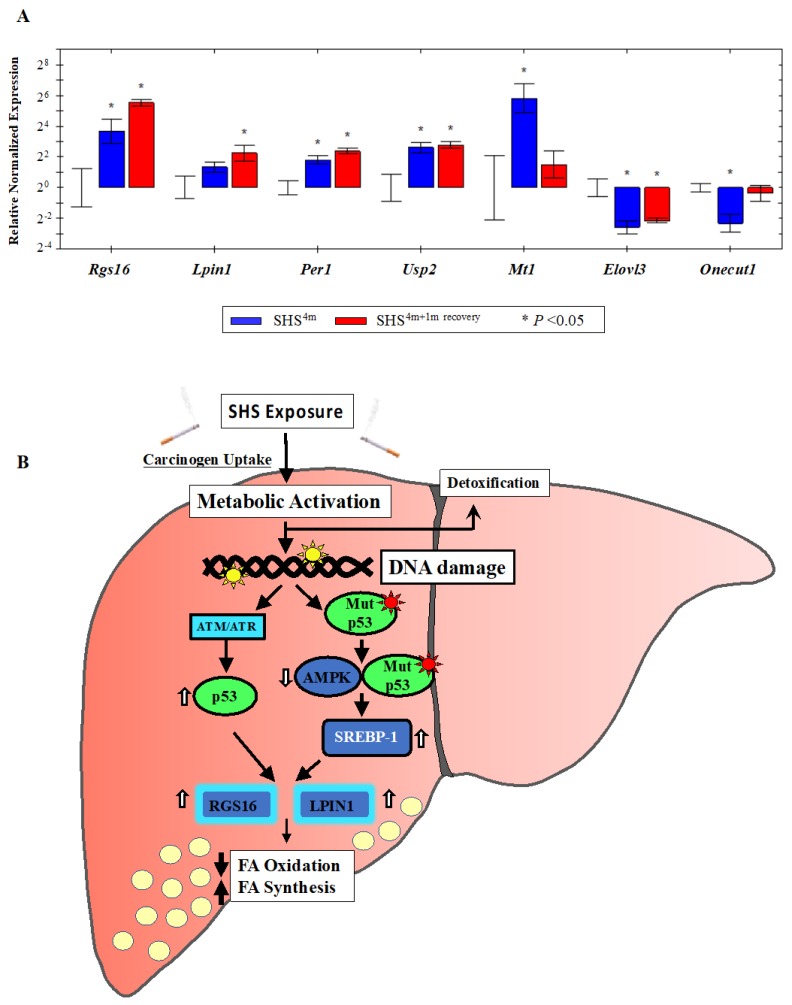
Gene validation by Reverse Transcription Quantitative PCR (RT-qPCR) and proposed model. (**A**) The expression status of individual gene targets identified by microarray analysis was examined by RT-qPCR. Bars represent the mean normalized gene expression (±SE) in SHS-exposed mice, before recovery (in blue) and after recovery (in red) relative to controls. All reactions (5 samples per experimental and control group) were performed in triplicate for a total of 15 reactions per biological set. Data were normalized using the endogenous housekeeping gene, glyceraldehyde-3-phosphate dehydrogenase (*Gapdh*), as reference. (**B**) Proposed model of SHS-induced hepatic fat accumulation through the involvement of wild-type p53 and/or gain-of-function p53 mutants. Vertical white arrows indicate up-regulation or down-regulation of target molecules; vertical black arrows show decrease in fatty acid (FA) oxidation or increase in fatty acid (FA) synthesis (see, text).

To validate the genome-wide gene expression data, we randomly selected several up-regulated or down-regulated targets from the 153 gene list (Table 1), and quantified the expression level of each gene by standard Reverse Transcription Quantitative PCR (RT-qPCR). Mean normalized expression levels of all selected genes in the SHS-exposed mice, before and after recovery, relative to age-matched controls are shown in Figure 6A. Consistent with the microarray data, RT-qPCR analysis of total RNA from the liver of SHS-exposed mice pre-recovery showed a ~13-fold increase in relative expression level of the regulator of G-protein signaling 16 gene (*Rgs16*). The expression level of *Rgs16* continued to increase in the SHS-exposed mice after one-month recovery, reaching ~46 times higher than that in age-matched controls (Figure 6A). The regulator of G-protein signaling 16 gene (*Rgs16*) is a key determinant of lipid metabolism and biosynthesis [43]. RGS16 has been shown to induce hepatic steatosis by inhibiting G_i_/G_q_-mediated fatty acid oxidation. Transgenic mice specifically expressing RGS16 protein in their hepatocytes have shown to have elevated levels of triglycerides and accumulation of fat deposits in their liver compared to control littermates, while *Rgs16* knockout mice have displayed the opposite phenotype [43].

Likewise, over-expression of the *Lipin1* (*Lpin1*) gene, which plays a crucial role in liver metabolism [44,45], was confirmed in the SHS-exposed mice by RT-qPCR analysis. LPIN1 is a bi-functional protein with distinct roles in lipid metabolism, depending on its subcellular localization [46]. In the nucleus, *Lpin1* interacts with the peroxisome proliferator-activated receptor α (PPARα) and PPARγ coactivator 1α (PGC-1α) to modulate the expression of genes involved in mitochondrial fatty acid oxidation [44]. In the cytoplasm, LPIN1 functions as a Mg^2+^-dependent phosphatidate phosphatase enzyme that catalyzes the conversion of phosphatidate to diacylglycerol, a key step in the biosynthesis of triacylglycerol [45]. As shown in Figure 6A, the expression level of *Lpin1* was increased in the SHS-exposed mice pre-recovery (2.5-fold) and continued to rise after one month of recovery (~5-fold) relative to age-matched controls. Of significance, both *Rgs16* and *Lpin1* were identified by IPA^®^ analysis as part of a subset of molecules affecting liver steatosis in the SHS-exposed mice (Figure 2A).

We also confirmed over-expression of the metallothionein 1 (*Mt1*) gene in the SHS-exposed mice before and after one-month recovery (Figure 6A). *Mt1* and its isoform *Mt2* belong to a family of small cysteine-rich and heavy metal binding proteins, the metallothioneins (MTs), that are involved in protective stress responses [47]. Synthesis of MTs has been reported to significantly increase due to a variety of stimuli, including oxidative stress, cytotoxicity, irradiation, and DNA damage [47]. Furthermore, we detected SHS-induced up-regulation of the ubiquitin specific peptidase 2 (*Usp2*) gene and its downstream target, the period circadian clock 1 (*Per1*) gene, in the SHS-exposed mice vs. controls. The transcription levels of these two genes were significantly elevated in the SHS-exposed mice, both before and after one-month recovery, as compared to age-matched controls (Figure 6A).

Moreover, we verified the SHS-induced down-regulation of other functionally important genes. The hepatocyte nuclear factor 6 (*Hnf6/Onecut1*) is a member of the *one cut* family of transcription factors, which modulates expression of numerous genes required for hepatocyte function. *Hfn6* is known to be down-regulated during liver injury [48]. The elongation of very long chain fatty acids (FEN1/Elo2, SUR4/Elo3, yeast)-like 3 (*Elovl3*) gene encodes a protein that plays a key role in elongation of long chain fatty acids, thus providing precursors for synthesis of sphingolipids and ceramides [49]. Down-regulation of *Onecut1* and *Elovl3* transcripts was confirmed in the SHS-exposed mice upon termination of exposure (0.20-fold and 0.17-fold, respectively) and remained persistent in the counterpart mice undergone one-month recovery in clean air (0.78-fold and 0.22-fold, respectively) (Figure 6A).

## 3. Discussion

First, we analyzed the hepatic transcriptome of SHS-exposed mice, pre- and post-recovery, using genome-wide gene expression analysis followed by functional network and molecular pathway analyses. As shown in Figure 1A and Table S1, SHS-exposure resulted in a significant transcriptomic response, with several hundred differentially expressed transcripts being detectable in the exposed mice immediately after treatment. One-month recovery in clean air only partially mitigated the SHS-induced transcriptional changes as the number of aberrant transcripts in the exposed mice undergone recovery remained substantially high (Figure 1A and Table S1). The persistent transcriptional changes in the SHS-exposed mice predominantly affected genes and functional networks involved in lipid metabolism and biosynthesis as well as in regulation of the endoplasmic reticulum where manufacturing of lipids occurs (Figure 2B, Figure S1 and Table 1). Of the common DEGs in the SHS-exposed mice pre- and post-recovery, 41% are known to modulate lipid metabolism, with 28% being specifically involved in the development of hepatic steatosis (Figure 2A and Table 1).

Upstream Regulator Analysis by IPA^®^ identified a complex network of eleven transcription factors and/or regulators (CHUK, IKBKG, IKBKB, SP1, STAT1, NFKBIA, FOXO3, NF-κB complex, RELA, NFKB1 and TP53) that are likely to account for the observed deregulation of lipid metabolism-specific genes and associated pathways in the SHS-exposed mice (Figure 3). This network includes NF-κB, whose activation in non-parenchymal cells is generally recognized to promote inflammation, fibrosis and hepatocarcinogenesis [37]. Of significance is also the predicted activation of TP53, a preferential target of DNA-damaging agents, such as tobacco smoke carcinogens [30,50,51], and a key regulator in fatty liver and insulin resistance [52]. TP53 interacts with the NK-kB complex, and crosstalk between the TP53 and NF-kB transcription factors has been shown to play a pivotal role in determining the cellular response to certain stimuli, e.g., DNA damage [53]. The predicted activation of TP53 is in accordance with the expression status of several downstream lipid targets found in the SHS-exposed mice, both before and after recovery in clean air (Figure 3).

A novel finding of our study is the SHS-induced up-regulation of *Rgs16* and *Lpin1*, two TP53 downstream effectors with crucial roles in lipid metabolism and liver steatosis (Figure 2A). RGS16 is known to be induced by doxorubicin in cells expressing wild-type p53 [54]. In normal lung fibroblasts, RGS16 is transcriptionally activated by exogenous expression of p53, either individually or in combination with retinoblastoma 1 [55]. TP53 can also up-regulate the expression of *Lpin1* via three p53 binding sites located on the first intron of the gene [56]. Whole body γ-irradiation of wild-type p53 mice, but not p53^−/−^ mice, has been shown to cause up-regulation of *Lpin 1* in several organs, with a pattern of expression resembling that of typical p53-responsive genes, including the *p21^WAF1^* gene [56]. In addition to being *bona fide* targets of p53, *Rgs16* and *Lpn1* contain potential sterol regulatory element (SRE) binding sites for SREBP-1, a key regulator of lipid metabolism under the negative control of AMPK [57].

A growing number of studies has shown a ‘noncanonical’ role for p53 in modulating lipid metabolism by either transcriptional regulation of target molecules involved in fatty acid synthesis and oxidation and lipid droplet formation or via direct protein-protein interactions [58,59,60,61]. Based on our results, we propose a model in which SHS induces TP53 via the DNA-damage response pathway, the ataxia–telangiectasia mutated/ataxia–telangiectasia and Rad3 related (ATM/ATR) kinase pathway). Active TP53, in turn, transcriptionally activates *Rgs16* and *Lpin1*, and most likely additional steatogenic genes in a tissue-specific context, ultimately leading to liver steatosis (Figure 6B). Alternatively, SHS can cause mutations in the *TP53* gene, and gain-of-function mutant forms of TP53 have been found to enhance fatty acid synthesis by inhibitory interaction with AMPKα, and consequent activation of SREBP-1 (Figure 6B) [60]. In turn, SREBP-1 can transcriptionally activate *Rgs16* and *Lpin1* through the SRE binding sites located on these genes. Yuan et al. have previously reported inactivation of AMPK and activation of SREBP-1c concurrent with hepatic lipid accumulation in mice fed with high-fat-diet and exposed to SHS [26]. Altogether, the deregulation of *Rgs16* and *Lpin1* in the SHS-exposed mice found in our study may provide novel insights into the interplay of carcinogen exposure, TP53-dependent response, and metabolic liver disease. Work in our laboratory is currently underway to further investigate the herein proposed model of SHS-induced hepatic steatosis via the TP53 pathway.

Lastly, the perturbation of key lipid genes in the SHS-exposed mice, which persisted after recovery in clean air, is highly consistent with the progressive accumulation of fat and other adverse histological changes observed in the liver of corresponding animals (Figure 4 and Figure 5). As shown in Figure 4A, the extent of fat accumulation in the liver of SHS-exposed mice progressively increased after recovery time in clean air (Figure 4A). Furthermore, SHS-exposed mice undergone recovery displayed more pronounced signs of liver injury, including disorganized lobular architecture, foci of inflammation, necrosis, and variable fibrosis (Figure 4B and Figure 5). One possible explanation for this observation is that the cascade of events triggered by exposure to SHS can further progress, even in the absence of SHS, and cause potentially irreversible liver injury. According to the ‘multiple-hit’ hypothesis, multiple events are required to promote NAFLD initiation and progression. Based on our results, SHS-induced disruption of lipid homeostasis with consequent steatosis may constitute the first hit. Additional factors (metabolic, environmental, genetic and/or epigenetic mechanisms) can further exacerbate liver injury mostly through modulation of pathways involved in mitochondrial dysfunction, oxidative stress, fatty acid biosynthesis, and inflammation, thus, leading to more severe forms of NAFLD. In other words, the first hit (SHS-induced liver steatosis) can increase the susceptibility to subsequent hits, and this could explain why we observed more pronounced effects in SHS^4m+1m recovery^ mice. Future follow up studies are needed to investigate the likelihood of the above scenarios in human populations.

## 4. Materials and Methods

### 4.1. Animal Care and Maintenance

This study was conducted in accordance with the recommendations described in the Guide for the Care and Use of Laboratory Animals of the National Institutes of Health, and all efforts were made to minimize animal suffering [62]. The study was approved by the Institutional Animal Care and Use Committee (IACUC) of the City of Hope (Protocol Number: 09012, 07 January 2009). All mice were fed a standard diet consisting, at a caloric level, of 25% proteins, 13% fat, and 62% carbohydrates (PicoLab^®^ Rodent Diet 20, PMI Nutrition International, LLC., Brentwood, MO, USA). At all times, including the exposure phase and recovery period, the mice had access to food and water ad libitum.

### 4.2. Smoking Machine and SHS Exposure

The smoking machine and exposure protocol have been described in detail in [29,30]. Briefly, SHS was generated using a custom-made TE-10 smoking machine (Teague Enterprises, Woodland, CA, USA). The TE-10 smoking machine is a microprocessor-controlled unit that can generate mainstream smoke, sidestream smoke, or a combination of the two. The machine was programmed to burn 3R4F Reference Kentucky cigarettes (Tobacco Research Institute, University of Kentucky, Lexington, KY, USA), and produce a mixture of sidestream smoke (89%) and mainstream smoke (11%). This formulation is conventionally used to mimic SHS for in vivo exposure and is representative of the SHS inhaled by humans in real life [32,63,64].

At the outset, all experimental mice underwent an acclimatization period during which they were gradually exposed ‘whole body’ to incremental doses of SHS. Following the acclimatization period, the mice were maintained on a SHS exposure regimen, which included 5 h per day, 5 days per week, and four-month whole body exposure to SHS, produced by continuous burning of 7–9 cigarettes. The average concentrations of total suspended particulate (TSP) in the exposure chambers were 233.0 ±15.4 mg/m^3^ at any given time during the four-month SHS exposure. The respective average TSP concentrations correspond to SHS generated through continuous smoking of 8.0 ± 0.5 cigarettes, at any given time during the four-month SHS exposure [29].

We note that whole body smoke exposure in rodents may result in residual transdermal and gastrointestinal absorption of smoke particles consequent to grooming [30]. However, ‘nose-only’ exposure can cause stress and discomfort for the animals, which would be pronounced in long-term studies, such as the present one. Therefore, we chose whole body exposure of mice to SHS based on tolerability and practicality of this approach and its compatibility with our study design. In addition, whole body smoke exposure in mice recapitulates real-life human exposure to SHS [30].

### 4.3. Study Design

Adult male mice (6–8 weeks old), on a C57BL/6 genetic background, were randomly assigned to two groups, including (1) ‘experimental’ (SHS exposure) and (2) ‘control’ (sham-treatment in clean air). The experimental group was divided in two subgroups (5 mice per subgroup), including four-month SHS exposure (SHS^4m^) and four-month SHS exposure plus one-month recovery in clean air (SHS^4m+1m recovery^). Age-matched control mice were similarly subdivided in sham-treatment subgroups, with and without recovery (5 mice per subgroup). The sham-treated mice were exposed to filtered high-efficiency particulate air (HEPA) in lieu of SHS, as described previously [29,30,31,32]. At the end of all experiments, the SHS-exposed and control mice were euthanized by CO_2_ asphyxiation and various tissues and organs, including the liver, were harvested and kept at −80 °C until further analysis. We note that based on life span, four-month SHS exposure in mice is equivalent to approximately 12 years human exposure to SHS, which is a realistic and biologically relevant exposure scenario in real-life. In our previously published studies [29,30,31,32,65,66], we have also verified that four-month SHS exposure is sufficient to elicit significant genotoxic, epigenetic, and transcriptomic responses in various organs and tissues of male C57BL/6 mice. We have also confirmed that five mice per group are sufficient to yield, at a minimum, a study power of 1 − β = 80%, and statistically significant results at *p* < 0.05.

### 4.4. Genome-Wide Gene Expression Analysis

To construct the hepatic transcriptome in SHS-exposed mice, we used the GeneChip*^®^* Mouse Genome 430 2.0 Array (originally from Affymetrix Inc., Santa Clara, CA, USA; currently Thermo Fisher Scientific, Waltham, MA, USA). This microarray platform enables interrogation of over 39,000 transcripts and variants from more than 34,000 well-characterized mouse genes. Briefly, total RNA was isolated from liver tissues of SHS-exposed mice and controls, using the RNeasy Mini Kit (Qiagen, Valencia, CA, USA). Synthesis of double-stranded cDNA from total RNA, fragmentation, hybridization, staining, and microarray scanning were performed according to the manufacturer’s instructions (Affymetrix Inc.). Quality control evaluation and processing and analysis of expression data were performed using the Affymetrix Expression Console™ software (Affymetrix Inc.). The Bioconductor package ‘ArrayTools’ was used to identify differentially expressed genes in experimental groups relative to controls, as described previously [67]. To establish gene expression trends within each experimental group, significant gene lists were examined by hierarchical clustering analysis and principal component analysis (PCA) using the Partek*^®^* Genomics Suite*^®^* software (Partek Incorporated, St. Louis, MO, USA). Raw microarray data have been deposited in the Gene Expression Omnibus database at NCBI (accession number: GSE139440; htttp://www.ncbi.nlm.nih.gov/geo/).

### 4.5. Gene Ontology and Canonical Pathways Analysis

Gene ontology (GO) analysis was performed using a combination of the Database for Annotation, Visualization and Integrated Discovery (DAVID) Bioinformatics Tool v.6.8 [68] and the Ingenuity*^®^* Pathway Analysis (IPA*^®^*) v.9 tool (QIAGEN Bioinformatics, Redwood City, CA, USA; www.qiagenbioinformatics.com). The Functional Clustering Analysis tool in DAVID was used to group together similar annotation terms for all categories, while functional identification of gene networks, canonical pathways, and upstream regulators was done by IPA*^®^*.

### 4.6. Histological Examination

For histological visualization of fat content and neutral triglycerides, we performed Oil Red O (ORO) staining on liver sections prepared from SHS-exposed mice and controls, according to a published protocol [69]. Bright-field images were captured with an Olympus microscope (Camera Model DP27, Tokyo, Japan), at several magnifications, using the CellSens Standard software (Olympus, Tokyo, Japan). Quantification of lipid droplets in the ORO-stained slides was achieved by measuring the area occupied by red pixels, in ImageJ software (https://imagej.nih.gov/ij/), as described previously [69].

Paraffin-embedded liver sections were stained with hematoxylin and eosin (H&E), Masson’s trichrome and Periodic Acid-Schiff (PAS) stain according to standard procedures [70,71]. Images were acquired with the Philips IntelliSite Pathology Solutions (PIPS) system.

### 4.7. Reverse Transcription Quantitative PCR (RT-qPCR)

For validation purposes, we used a standard RT-qPCR protocol [66] to determine the expression level of single up-regulated or down-regulated genes identified by microarray analysis. Detailed descriptions for RT-qPCR method are available in Supplementary Material.

## 5. Conclusions

We have demonstrated, for the first time, that subchronic exposure of mice to SHS, independently of diet, induces liver steatosis by modulating genes and functional pathways involved in lipid metabolism. Our findings underscore how an environmental carcinogen, such as SHS, in addition to cancer-causing effects, may contribute to other adverse health consequences, specifically metabolic liver disease.

## Figures and Tables

**Table 1:** List of differentially expressed genes (*n* = 153) identified in the liver of SHS-exposed mice, both before and after recovery time, relative to controls.

Expression ^1^ Log Ratio	ID	D ^2^	Symbol ^3^	Entrez Gene Name	Location
3.543	1426037_a_at	D	RGS16 *	regulator of G-protein signaling 16	Cytoplasm
3.466	1422557_s_at	D	***Mt1***	metallothionein 1	Cytoplasm
3.26	1428942_at		***Mt2***	metallothionein 2	Other
3.245	1417168_a_at	D	*USP2*	ubiquitin specific peptidase 2	Cytoplasm
2.943	1422257_s_at	D	***CYP2B6***	cytochrome P450 family 2 subfamily B member 6	Cytoplasm
2.387	1442025_a_at	D	***ZBTB16***	zinc finger and BTB domain containing 16	Nucleus
2.265	1418288_at	D	LPIN1 *	lipin 1	Nucleus
2.221	1427747_a_at		***LCN2***	lipocalin 2	Extracellular Space
2.174	1428223_at		MFSD2A *	major facilitator superfamily domain containing 2A	Plasma Membrane
2.168	1443147_at	D	ACOT1 *	acyl-CoA thioesterase 1	Cytoplasm
2.095	1416125_at		*FKBP5*	FK506 binding protein 5	Nucleus
1.956	1435188_at		*CIART*	circadian associated repressor of transcription	Nucleus
1.933	1425837_a_at		NOCT *	nocturnin	Nucleus
1.922	1451190_a_at	D	*SBK1*	SH3 domain binding kinase 1	Other
1.87	1428923_at		***PPP1R3G***	protein phosphatase 1 regulatory subunit 3G	Cytoplasm
1.864	1451548_at	D	*UPP2*	uridine phosphorylase 2	Cytoplasm
1.774	1460241_a_at	D	***ST3GAL5***	ST3 beta-galactoside alpha-2,3-sialyltransferase 5	Cytoplasm
1.759	1419590_at		*Cyp2b13/Cyp2b9*	cytochrome P450, family 2, subfamily b, polypeptide 9	Cytoplasm
1.707	1416432_at		***PFKFB3***	6-phosphofructo-2-kinase/fructose-2,6-biphosphatase 3	Cytoplasm
1.61	1429144_at	D	*GPCPD1*	glycerophosphocholine phosphodiesterase 1	Cytoplasm
1.598	1434437_x_at	D	*RRM2*	ribonucleotide reductase regulatory subunit M2	Nucleus
1.594	1416933_at		POR *	cytochrome p450 oxidoreductase	Cytoplasm
1.542	1439489_at		FFAR4 *	free fatty acid receptor 4	Plasma Membrane
1.542	1448162_at		***VCAM1***	vascular cell adhesion molecule 1	Plasma Membrane
1.523	1429206_at		*RHOBTB1*	Rho related BTB domain containing 1	Other
1.518	1453023_at		*ANKHD1/ANKHD1-EIF4EBP3*	ankyrin repeat and KH domain containing 1	Other
1.493	1434473_at		*SLC16A5*	solute carrier family 16 member 5	Plasma Membrane
1.492	1417761_at	D	***APOA4***	apolipoprotein A4	Extracellular Space
1.469	1417602_at		***PER2***	period circadian clock 2	Nucleus
1.45	1425824_a_at		*PCSK4*	proprotein convertase subtilisin/kexin type 4	Extracellular Space
1.437	1448239_at		HMOX1 *	heme oxygenase 1	Cytoplasm
1.433	1452416_at		*IL6R*	interleukin 6 receptor	Plasma Membrane
1.418	1427473_at		*Gstm3*	glutathione S-transferase, mu 3	Cytoplasm
1.403	1449851_at		***PER1***	period circadian clock 1	Nucleus
1.38	1417904_at		*DCLRE1A*	DNA cross-link repair 1A	Nucleus
1.336	1450505_a_at	D	*FAM134B*	family with sequence similarity 134, member B	Cytoplasm
1.334	1440840_at		*SLC16A7*	solute carrier family 16 member 7	Plasma Membrane
1.302	1423233_at		***CEBPD***	CCAAT/enhancer binding protein delta	Nucleus
1.296	1416773_at		*WEE1*	WEE1 G2 checkpoint kinase	Nucleus
1.279	1435459_at	D	*FMO2*	flavin containing monooxygenase 2	Cytoplasm
1.279	1426452_a_at		*RAB30*	RAB30, member RAS oncogene family	Cytoplasm
1.268	1421681_at	D	*NRG4*	neuregulin 4	Extracellular Space
1.259	1435495_at		ADORA1 *	adenosine A1 receptor	Plasma Membrane
1.251	1426850_a_at		***MAP2K6***	mitogen-activated protein kinase kinase 6	Cytoplasm
1.247	1421852_at		*KCNK5*	potassium two pore domain channel subfamily K member 5	Plasma Membrane
1.246	1443870_at		***ABCC4***	ATP binding cassette subfamily C member 4	Plasma Membrane
1.242	1422230_s_at		***CYP2A6 (includes others)***	cytochrome P450 family 2 subfamily A member 6	Cytoplasm
1.239	1458442_at		*AI132709*	expressed sequence AI132709	Other
1.238	1424175_at		***TEF***	TEF, PAR bZIP transcription factor	Nucleus
1.231	1424744_at		*SDS*	serine dehydratase	Cytoplasm
1.213	1434292_at		*Snhg11*	small nucleolar RNA host gene 11	Other
1.212	1418780_at		***CYP39A1***	cytochrome P450 family 39 subfamily A member 1	Cytoplasm
1.207	1453410_at		***ANGPTL4***	angiopoietin like 4	Extracellular Space
1.199	1456156_at		***LEPR***	leptin receptor	Plasma Membrane
1.198	1449565_at		***Cyp2g1***	cytochrome P450, family 2, subfamily g, polypeptide 1	Cytoplasm
1.198	1449498_at		*MARCO*	macrophage receptor with collagenous structure	Plasma Membrane
1.197	1428352_at		*ARRDC2*	arrestin domain containing 2	Other
1.185	1418595_at		*PLIN4*	perilipin 4	Cytoplasm
1.178	1417042_at		***SLC37A4***	solute carrier family 37 member 4	Cytoplasm
1.169	1426980_s_at		*EPOP*	elongin BC and polycomb repressive complex 2 associated protein	Other
1.164	1445574_at	D	PPARGC1B *	PPARG coactivator 1 beta	Nucleus
1.162	1429809_at		*TMTC2*	transmembrane and tetratricopeptide repeat containing 2	Cytoplasm
1.152	1455958_s_at		*PPTC7*	PTC7 protein phosphatase homolog	Cytoplasm
1.151	1431339_a_at	D	*EFHD2*	EF-hand domain family member D2	Other
1.142	1428512_at		*BHLHB9*	basic helix-loop-helix domain containing, class B, 9	Cytoplasm
1.141	1455002_at	D	*PTP4A1*	protein tyrosine phosphatase type IVA, member 1	Cytoplasm
1.133	1428926_at	D	*FBXO31*	F-box protein 31	Extracellular Space
1.123	1416286_at		***RGS4***	regulator of G-protein signaling 4	Cytoplasm
1.115	1432543_a_at		*KLF13*	Kruppel like factor 13	Nucleus
1.111	1434456_at		*RUNDC3B*	RUN domain containing 3B	Other
1.108	1435860_at		*SLC5A6*	solute carrier family 5 member 6	Plasma Membrane
1.102	1427912_at		*CBR3*	carbonyl reductase 3	Cytoplasm
1.098	1456395_at	D	PPARGC1A *	PPARG coactivator 1 alpha	Nucleus
1.091	1454799_at		***GPAT3***	glycerol-3-phosphate acyltransferase 3	Cytoplasm
1.047	1419758_at		***ABCB1***	ATP binding cassette subfamily B member 1	Plasma Membrane
1.04	1423627_at		***NQO1***	NAD(P)H quinone dehydrogenase 1	Cytoplasm
1.036	1438211_s_at		***DBP***	D-box binding PAR bZIP transcription factor	Nucleus
1.032	1424815_at		*GYS2*	glycogen synthase 2	Cytoplasm
1.021	1448568_a_at		*SLC20A1*	solute carrier family 20 member 1	Plasma Membrane
1.007	1428487_s_at	D	*COQ10B*	coenzyme Q10B	Cytoplasm
1.007	1420772_a_at		TSC22D3 *	TSC22 domain family member 3	Nucleus
1.006	1433816_at		*SLC25A51*	solute carrier family 25 member 51	Cytoplasm
−1.003	1439377_x_at		*CDC20*	cell division cycle 20	Nucleus
−1.006	1455293_at		*LEO1*	LEO1 homolog, Paf1/RNA polymerase II complex component	Nucleus
−1.008	1431056_a_at		LPL *	lipoprotein lipase	Cytoplasm
−1.013	1450010_at		***HSD17B12***	hydroxysteroid 17-beta dehydrogenase 12	Cytoplasm
−1.022	1417292_at		*Ifi47*	interferon gamma inducible protein 47	Cytoplasm
−1.022	1417792_at		*ZNF638*	zinc finger protein 638	Nucleus
−1.027	1452445_at		*SLC41A2*	solute carrier family 41 member 2	Plasma Membrane
−1.036	1448986_x_at		*DNASE2*	deoxyribonuclease 2, lysosomal	Cytoplasm
−1.037	1436931_at		*RFX4*	regulatory factor X4	Nucleus
−1.039	1450035_a_at	D	*PRPF40A*	pre-mRNA processing factor 40 homolog A	Nucleus
−1.04	1428022_at		OBP2B *	odorant binding protein 2B	Extracellular Space
−1.042	1427356_at		*FAM89A*	family with sequence similarity 89 member A	Other
−1.042	1424033_at		*SRSF7*	serine and arginine rich splicing factor 7	Nucleus
−1.043	1457758_at		*ENY2*	ENY2, transcription and export complex 2 subunit	Nucleus
−1.045	1416403_at		***ABCB10***	ATP binding cassette subfamily B member 10	Cytoplasm
−1.047	1450846_at		*BZW1*	basic leucine zipper and W2 domains 1	Cytoplasm
−1.048	1438713_at		*RASSF8*	Ras association domain family member 8	Extracellular Space
−1.051	1433515_s_at	D	***ETNK1***	ethanolamine kinase 1	Cytoplasm
−1.057	1437864_at		ADIPOR2 *	adiponectin receptor 2	Plasma Membrane
−1.057	1451122_at	D	***IDI1***	isopentenyl-diphosphate delta isomerase 1	Cytoplasm
−1.061	1425206_a_at		*UBE3A*	ubiquitin protein ligase E3A	Nucleus
−1.073	1420379_at		***Slco1a1***	solute carrier organic anion transporter family, member 1a1	Plasma Membrane
−1.077	1448183_a_at		***HIF1A***	hypoxia inducible factor 1 alpha subunit	Nucleus
−1.085	1452030_a_at		*HNRNPR*	heterogeneous nuclear ribonucleoprotein R	Nucleus
−1.085	1428372_at		*ST5*	suppression of tumorigenicity 5	Cytoplasm
−1.086	1450484_a_at		*CMPK2*	cytidine/uridine monophosphate kinase 2	Cytoplasm
−1.091	1431024_a_at		*ARID4B*	AT-rich interaction domain 4B	Nucleus
−1.108	1417832_at		*SMC1A*	structural maintenance of chromosomes 1A	Nucleus
−1.109	1424842_a_at		*ARHGAP24*	Rho GTPase activating protein 24	Cytoplasm
−1.11	1455324_at	D	*PLCXD2*	phosphatidylinositol specific phospholipase C X domain containing 2	Other
−1.12	1426458_at		*SLMAP*	sarcolemma associated protein	Plasma Membrane
−1.128	1438269_at		*ZBTB38*	zinc finger and BTB domain containing 38	Nucleus
−1.131	1449931_at		*CPEB4*	cytoplasmic polyadenylation element binding protein 4	Plasma Membrane
−1.136	1442537_at		*CYP2U1*	cytochrome P450 family 2 subfamily U member 1	Cytoplasm
−1.158	1449854_at		***NR0B2***	nuclear receptor subfamily 0 group B member 2	Nucleus
−1.16	1427574_s_at		*SH3D19*	SH3 domain containing 19	Plasma Membrane
−1.165	1435775_at		***CLOCK***	clock circadian regulator	Nucleus
−1.169	1429772_at	D	*PLXNA2*	plexin A2	Plasma Membrane
−1.171	1437932_a_at		*CLDN1*	claudin 1	Plasma Membrane
−1.172	1423325_at		*PNN*	pinin, desmosome associated protein	Plasma Membrane
−1.18	1447927_at	D	*GBP6*	guanylate binding protein family member 6	Cytoplasm
−1.205	1425099_a_at		ARNTL *	aryl hydrocarbon receptor nuclear translocator like	Nucleus
−1.208	1444512_at		*ARHGAP29*	Rho GTPase activating protein 29	Cytoplasm
−1.238	1442367_at		***ATP11C***	ATPase phospholipid transporting 11C	Plasma Membrane
−1.27	1417982_at		INSIG2 *	insulin induced gene 2	Cytoplasm
−1.275	1427513_at		*BC024137*	cDNA sequence BC024137	Other
−1.276	1430896_s_at		***NUDT7***	nudix hydrolase 7	Cytoplasm
−1.289	1450090_at		*Zfp101*	zinc finger protein 101	Nucleus
−1.295	1449514_at		***GRK5***	G protein-coupled receptor kinase 5	Plasma Membrane
−1.325	1421092_at		***SERPINA12***	serpin family A member 12	Cytoplasm
−1.343	1423571_at		***S1PR1***	sphingosine-1-phosphate receptor 1	Plasma Membrane
−1.346	1420531_at		***Hsd3b4 (includes others)***	hydroxy-delta-5-steroid dehydrogenase, 3 beta- and steroid delta-isomerase 4	Cytoplasm
−1.354	1422769_at	D	*SYNCRIP*	synaptotagmin binding cytoplasmic RNA interacting protein	Nucleus
−1.379	1437581_at		*ZNF800*	zinc finger protein 800	Other
−1.395	1430785_at	D	*SDR9C7*	short chain dehydrogenase/reductase family 9C member 7	Other
−1.402	1426215_at		DDC *	dopa decarboxylase	Cytoplasm
−1.405	1426645_at		*HSP90AA1*	heat shock protein 90 alpha family class A member 1	Cytoplasm
−1.434	1433446_at	D	*HMGCS1*	3-hydroxy-3-methylglutaryl-CoA synthase 1	Cytoplasm
−1.472	1423397_at		*UGT2B28*	UDP glucuronosyltransferase family 2, member B28	Cytoplasm
−1.482	1424709_at	D	***SC5D***	sterol-C5-desaturase	Cytoplasm
−1.509	1450264_a_at		***CHKA***	choline kinase alpha	Cytoplasm
−1.554	1438751_at		*SLC30A10*	solute carrier family 30 member 10	Other
−1.573	1417065_at		***EGR1***	early growth response 1	Nucleus
−1.58	1433944_at		*HECTD2*	HECT domain E3 ubiquitin protein ligase 2	Cytoplasm
−1.622	1431817_at		*Adh6-ps1*	alcohol dehydrogenase 6 (class V), pseudogene 1	Other
−1.663	1439300_at	D	*CHIC1*	cysteine rich hydrophobic domain 1	Plasma Membrane
−1.666	1427347_s_at	D	*TUBB2A*	tubulin beta 2A class IIa	Cytoplasm
−1.692	1450018_s_at	D	*SLC25A30*	solute carrier family 25 member 30	Cytoplasm
−1.896	1448092_x_at	D	*Serpina4-ps1*	serine (or cysteine) peptidase inhibitor, clade A, member 4, pseudogene 1	Other
−2.034	1420722_at		***ELOVL3***	ELOVL fatty acid elongase 3	Cytoplasm
−2.382	1421447_at	D	***ONECUT1***	one cut homeobox 1	Nucleus

^1^ A “positive” (+) fold ratio indicates up-regulation while a “negative” (−) fold ratio indicates down-regulation; ^2^ D, duplicate transcripts were identified for that gene; ^3^ genes involved in lipid metabolism (*n* = 63) are indicated in bold. The asterisk (*) marks genes known to play a role in liver steatosis.

## Supplementary Materials

The following are available online at https://www.mdpi.com/1422-0067/21/4/1296/s1.

## Abbreviations

AMPK	AMP-activated kinase
APR	Acute Phase Response
CHUK	Component of inhibitor of nuclear factor kappa B kinase complex
DAVID	Database for Annotation, Visualization and Integrated Discovery
DEGs	Differentially expressed genes
*Elovl3*	Elongation of very long chain fatty acids (FEN1/Elo2, SUR4/Elo3, yeast)-like 3
FOXO3	Forkhead box O3
GO	Gene Ontology
*Gapdh*	Glyceraldehyde-3-phosphate dehydrogenase
H&E	hematoxylin and eosin
*Hmox1*	Heme oxygenase 1
*Hnf6/Onecut1*	Hepatocyte nuclear factor 6
HEPA	High-efficiency particulate air
IPA	Ingenuity Pathway Analysis
IKBKB	Inhibitor of nuclear factor kappa B kinase subunit beta
IKBKG	Inhibitor of nuclear factor kappa B kinase regulatory subunit gamma
IACUC	Institutional Animal Care and Use Committee
IGF	Insulin-like growth factor
IL-1	interleukin-1
*Lpin1*	Lipin 1
LPS	lipopolysaccharide
NAFLD	Non-alcoholic fatty liver disease
MTs	Metallothioneins
*Mt1*	Metallothionein 1
NFKBIA	NFKB inhibitor alpha
NF-κB	Nuclear factor kappa B
NFKB1	Nuclear factor kappa B subunit 1
ORO	Oil Red O
PAS	Periodic Acid-Schiff
PCA	principal component analysis
PPARα	Peroxisome proliferator-activated receptor α
PGC-1α	PPARγ coactivator 1α
RELA	RELA proto-oncogene
RXR	NF-κB subunit Retinoid X Receptor
*Per1*	Period circadian clock 1
SHS	Secondhand smoke
ROS	Reactive oxygen species
*Rgs16*	Regulator of G-protein signaling 16
RT-qPCR	Reverse transcription quantitative polymerase chain reaction
SP1	Sp1 transcription factor
STAT1	Signal transducer and activator of transcription 1
SREBPs	Sterol regulatory elements binding sites for proteins
SREBP-1c	Sterol regulatory element binding protein-1c
TSP	Total suspended particulate
*Tsc22d3*	TSC22 domain family, member 3
TP53	Tumor protein p53
*Usp2*	Ubiquitin specific peptidase 2
