# Supplementary material for: Secondhand Smoke Induces Liver Steatosis through Deregulation of Genes Involved in Hepatic Lipid Metabolism
Source: Int J Mol Sci. 2020 Feb 14;21(4):1296. doi: 10.3390/ijms21041296 (PMC7072934; doi:10.3390/ijms21041296)
Supplement: Supplementary file 1 [file ijms-21-01296-s001.pdf]

SUPPORTING DOCUMENTS

Manuscript title: *Secondhand smoke induces liver steatosis through deregulation of genes involved in hepatic lipid metabolism*

Authors: Stella Tommasi, Jae-In Yoon, and Ahmad Besaratinia

Content:

- Supplemental Materials
- Supplemental Figure Legends
- Supplemental Figure 1 and Figure 2
- Supplemental Table 1

SUPPLEMENTAL MATERIALS

Genome-wide gene expression analysis

To construct the hepatic transcriptome in SHS-exposed mice, we used the GeneChip® Mouse Genome 430 2.0 Array (originally from Affymetrix Inc., Santa Clara, CA; currently Thermo Fisher Scientific, Waltham, MA). This microarray platform enables interrogation of over 39,000 transcripts and variants from more than 34,000 well-characterized mouse genes. Briefly, total RNA was isolated from mouse liver tissues using the RNeasy Mini Kit (Qiagen, Valencia, CA). Before microarray analysis, RNA samples were checked for quality control using the RNA 6000 Nano Chip kit in an Agilent 2100 Bioanalyzer (Agilent Technologies, Santa Clara, CA). Synthesis of double-stranded cDNA from total RNA, fragmentation, hybridization, staining, and microarray scanning were performed according to the manufacturer's instructions. Quality control evaluation, processing and analysis of the gene expression data was performed using the Affymetrix Expression Console™ software (Affymetrix Inc.). The Bioconductor package 'ArrayTools' was then used to identify differentially expressed genes between various experimental groups as compared to non-treated control, based on a cutoff P value of <0.05, and a two-fold change in the level of expression. To establish gene expression trends within each experimental group as well as across all groups, significant gene lists were examined by hierarchical clustering analysis and principal component analysis (PCA) using the Partek® Genomics Suite® software (Partek Incorporated, St. Louis, MO).

Reverse transcription quantitative PCR (RT-qPCR)

Total RNA (0.5 μg) from mouse liver was treated with DNase I and reverse transcribed into cDNA using the iScript™ Reverse Transcription Supermix (Bio-Rad laboratories, Inc., Hercules, CA).

The synthesized cDNA (10 ng) was then PCR amplified using gene-specific primers and the SsoAdvanced™ Universal SYBR® Green Supermix (Bio-Rad laboratories, Inc., Hercules, CA). The mouse glyceraldehyde-3-phosphate dehydrogenase (*Gapdh*) was used as a reference gene. All PCR reactions were carried out using the CFX96 Touch™ Real-Time PCR detection system (Bio-Rad Laboratories, Hercules, CA). The cycling conditions included a pre-incubation step at 95°C for 2 minutes, followed by forty cycles at 95°C for 5 seconds and 58°C for 30 seconds. All reactions (5 samples per group) were performed in triplicate for a total of 15 reactions per biological set. Fold changes in the transcript levels were calculated in the biological sets (i.e., experimental versus control) using the Bio-Rad CFX Maestro™ software (Bio-Rad Laboratories, Hercules, CA). The primer sets used for RT-qPCR are available upon request.

SUPPLEMENTAL FIGURE LEGENDS

Supplemental Figure 1. Functional network analysis of aberrantly expressed genes in SHS exposed mice. The 153-gene list was imported into IPA® software and functional network analysis was performed. The top impacted networks are illustrated and show extensive involvement of genes relevant to lipid metabolism and biosynthesis. Other relevant gene networks included molecules implicated in behavior and nervous system development and function, cell death and survival, drug metabolism and small molecule biochemistry. Red and green nodes represent upregulated and down-regulated genes, respectively. The intensity of the red and green color indicates the level of up-regulation and down-regulation, respectively. White nodes show molecules that are not included in the datasets but interact with other components of the network. Solid and dotted lines indicate a direct or indirect relationship, respectively, among molecules.

Supplemental Figure 2. Chart of mice body weights. (A) Control mice (sham-exposure in clean air). (B) 4-month SHS-exposed mice, during treatment and after recovery time in clean air. Results are expressed as medians + 95% CIs.

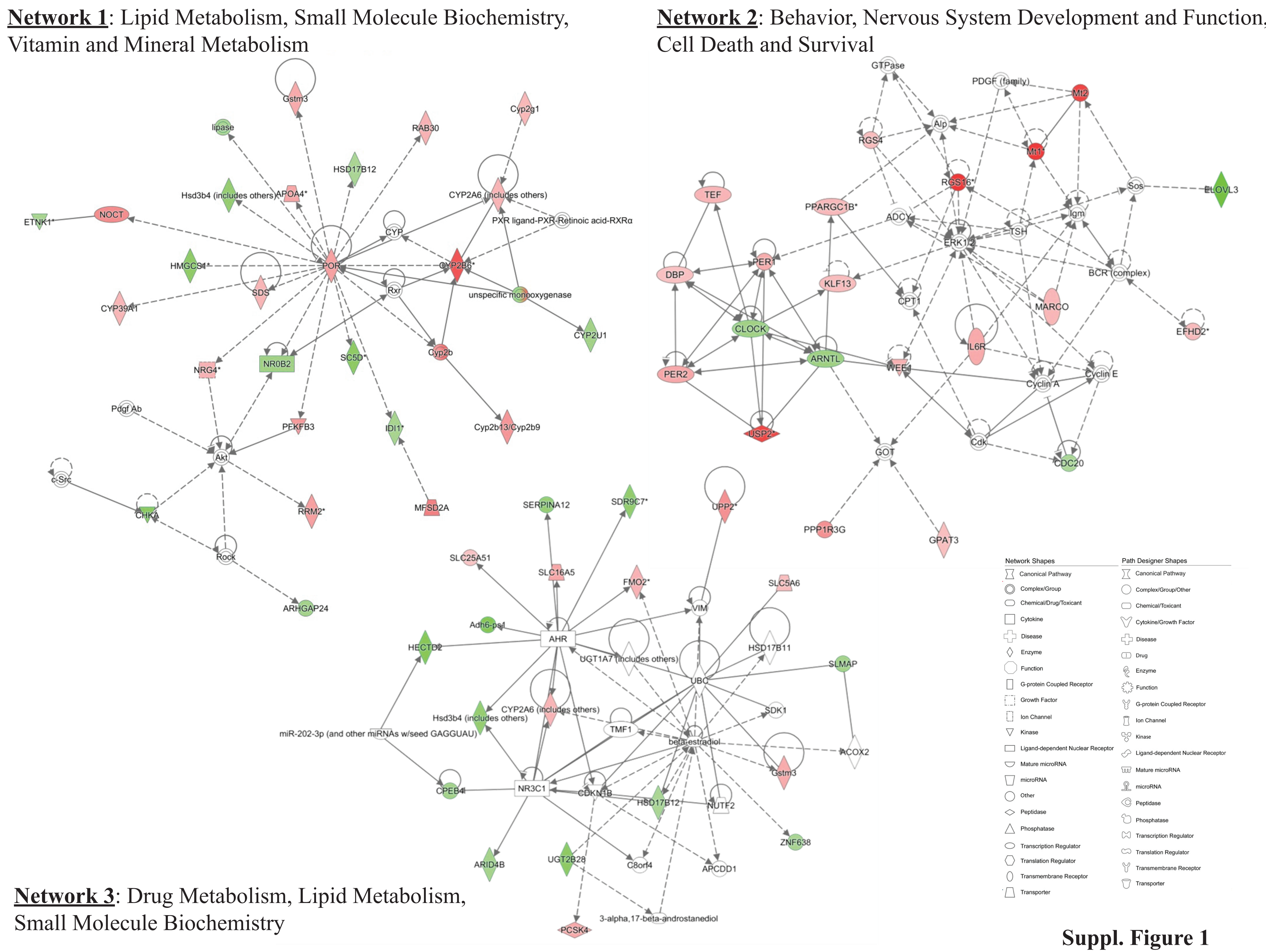

Suppl. Figure 1

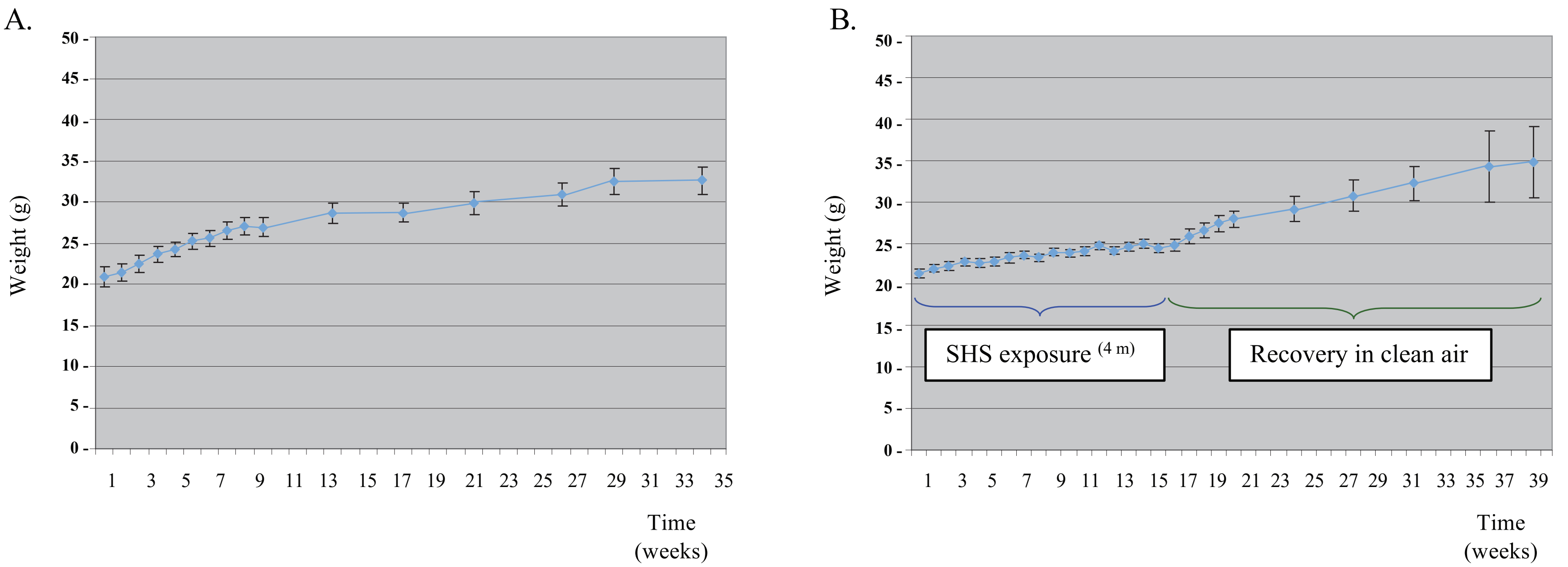

Suppl. Figure 2

Supplemental Table 1: Compiled lists of aberrant transcripts identified by microarray analysis in the various contrast groups.

SHS4 vs C

	Probeset_id	Log2Ratio.1	P.Value	Adj.P.Value	Symbol	Description	Chromosome	GenBank
1	1428942_at	5.84352	4.05E-13	2.37E-09	Mt2	metallothionein 2	8	AA796766
2	1422557_s_at	5.30464	2.27E-09	9.10E-07	Mt1	metallothionein 1	8	NM_013602
3	1422257_s_at	4.29557	1.21E-09	5.82E-07	Cyp2b10	cytochrome P450, family 2, subfamily b, polypeptide 10	7	NM_009998
4	1425645_s_at	4.21353	8.17E-10	4.64E-07	Cyp2b10	cytochrome P450, family 2, subfamily b, polypeptide 10	7	AF128849
5	1427747_a_at	3.97183	3.29E-06	0.000156818	Lcn2	lipocalin 2	2	X14607
6	1451787_at	3.89116	4.60E-10	3.41E-07	Cyp2b10	cytochrome P450, family 2, subfamily b, polypeptide 10	7	AF128849
7	1442025_a_at	3.46291	1.51E-05	0.000444652				AI467657
8	1418918_at	3.35596	3.07E-05	0.000745593	Igfbp1	insulin-like growth factor binding protein 1	11	NM_008341
9	1417168_a_at	3.32643	3.05E-08	6.12E-06	Usp2	ubiquitin specific peptidase 2	9	AI553394
10	1419874_x_at	3.24507	9.63E-06	0.000331041	Zbtb16	zinc finger and BTB domain containing 16	9	AA419994
11	1426037_a_at	2.97804	0.000111751	0.00186664	Rgs16	regulator of G-protein signaling 16	1	U94828
12	1417169_at	2.94705	5.70E-09	1.84E-06	Usp2	ubiquitin specific peptidase 2	9	AI553394
13	1439489_at	2.9461	8.04E-14	1.04E-09	Gpr120	G protein-coupled receptor 120	19	AV025152
14	1416125_at	2.77194	8.03E-10	4.64E-07	Fkbp5	FK506 binding protein 5	17	U16959
15	1419149_at	2.76286	0.00135917	0.0116112	Serpine1	serine (or cysteine) peptidase inhibitor, clade E, member 1	5	NM_008871
16	1427473_at	2.75153	1.07E-13	1.04E-09	Gstm3	glutathione S-transferase, mu 3	3	J03953
17	1420438_at	2.72089	3.31E-06	0.000157	Orm2	orosomucoid 2	4	NM_011016
18	1428223_at	2.60089	5.01E-06	0.000211042	Mfsd2a	major facilitator superfamily domain containing 2A	4	AK006096
19	1418288_at	2.59881	9.09E-06	0.000318716	Lpin1	lipin 1	12	NM_015763
20	1460241_a_at	2.58887	7.52E-09	2.25E-06	St3gal5	ST3 beta-galactoside alpha-2,3-sialyltransferase 5	6	BB829192
21	1443137_at	2.58277	6.76E-08	1.07E-05				BB534298
22	1442026_at	2.50094	1.07E-05	0.000356174				AI467657
23	1433966_x_at	2.49832	0.000588845	0.00624027	Asns	asparagine synthetase	6	AV212753
24	1451204_at	2.47248	8.27E-06	0.000298352	Scara5	scavenger receptor class A, member 5 (putative)	14	BC016096
25	1426516_a_at	2.44265	1.71E-05	0.000485608	Lpin1	lipin 1	12	AK014526
26	1455265_a_at	2.44113	4.82E-05	0.00102418	Rgs16	regulator of G-protein signaling 16	1	BB100249
27	1419590_at	2.41136	0.00127051	0.0110635				NM_010000
28	1439163_at	2.36604	2.81E-05	0.000698778	Zbtb16	zinc finger and BTB domain containing 16	9	BQ174973
29	1434202_a_at	2.34657	7.14E-05	0.00136919	Fam107a	family with sequence similarity 107, member A	14	BF682848
30	1428306_at	2.33114	0.000341604	0.00422706	Ddit4	DNA-damage-inducible transcript 4	10	AK017926
31	1427474_s_at	2.28106	1.73E-13	1.27E-09	Gstm3	glutathione S-transferase, mu 3	3	J03953
32	1451190_a_at	2.24992	5.16E-09	1.72E-06	Sbk1	SH3-binding kinase 1	7	BC025837
33	1451612_at	2.23894	5.87E-11	8.59E-08	Mt1	metallothionein 1	8	BC027262

34	1429144_at	2.23539	2.35E-06	0.000124092	Gpcpd1	glycerophosphocholine phosphodiesterase GDE1 homolog (S. cerevisiae)	2	AV291259
35	1425837_a_at	2.20967	0.00027494	0.00360299				AF199491
36	1449198_a_at	2.17858	1.32E-09	6.05E-07	St3gal5	ST3 beta-galactoside alpha-2,3-sialyltransferase 5	6	BB829192
37	1453410_at	2.17309	2.24E-07	2.40E-05	Angptl4	angiopoietin-like 4	17	AK014564
38	1434437_x_at	2.16912	5.03E-07	4.13E-05	Rrm2	ribonucleotide reductase M2	12	AV301324
39	1419857_at	2.14086	1.51E-06	9.19E-05				AA254866
40	1448239_at	2.11829	3.52E-07	3.26E-05	Hmox1	heme oxygenase (decycling) 1	8	NM_010442
41	1424744_at	2.10163	6.61E-10	4.21E-07	Sds	serine dehydratase	5	BC021950
42	1450505_a_at	2.09765	2.05E-11	4.43E-08	Fam134b	family with sequence similarity 134, member B	15	NM_025459
43	1451548_at	2.08634	4.55E-06	0.000197361	Upp2	uridine phosphorylase 2	2	BC027189
44	1453023_at	2.07393	6.69E-08	1.07E-05				AK003441
45	1434473_at	2.0467	2.57E-05	0.00065555	Slc16a5	solute carrier family 16 (monocarboxylic acid transporters), member 5	11	AI647939
46	1427912_at	2.04574	1.87E-09	7.93E-07	Cbr3	carbonyl reductase 3	16	AK003232
47	1426452_a_at	2.03623	4.68E-05	0.00100547	Rab30	RAB30, member RAS oncogene family	7	BG070713
48	1435188_at	2.03088	1.09E-06	7.30E-05	Gm129	predicted gene 129	3	BB407125
49	1428923_at	2.02896	0.000408536	0.00479619	Ppp1r3g	protein phosphatase 1, regulatory (inhibitor) subunit 3G	13	AK005570
50	1423978_at	2.01376	1.63E-09	7.15E-07	Sbk1	SH3-binding kinase 1	7	BC025837
51	1460059_at	2.01241	8.24E-10	4.64E-07	Upp2	uridine phosphorylase 2	2	BB272732
52	1451095_at	1.99085	9.74E-05	0.00169291	Asns	asparagine synthetase	6	BC005552
53	1424969_s_at	1.98321	2.57E-06	0.000132034	Upp2	uridine phosphorylase 2	2	BC027189
54	1448162_at	1.98316	1.09E-08	2.94E-06	Vcam1	vascular cell adhesion molecule 1	3	BB250384
55	1435495_at	1.97942	1.29E-09	6.05E-07	Adora1	adenosine A1 receptor	1	BE630294
56	1423233_at	1.95653	1.77E-07	2.00E-05	Cebpd	CCAAT/enhancer binding protein (C/EBP), delta	16	BB831146
57	1455958_s_at	1.9231	7.21E-12	2.35E-08	Pptc7	PTC7 protein phosphatase homolog (S. cerevisiae)	5	AI881989
58	1416286_at	1.92027	4.05E-07	3.54E-05	Rgs4	regulator of G-protein signaling 4	1	NM_009062
59	1448226_at	1.91427	1.46E-05	0.000437558	Rrm2	ribonucleotide reductase M2	12	NM_009104
60	1417761_at	1.91092	6.88E-08	1.08E-05	Apoa4	apolipoprotein A-IV	9	BC010769
61	1416933_at	1.90963	6.80E-11	9.48E-08	Por	P450 (cytochrome) oxidoreductase	5	NM_008898
62	1450611_at	1.90332	1.11E-05	0.000364548	Orm3	orosomucoid 3	4	NM_013623
63	1452426_x_at	1.882	6.01E-05	0.00121616				BC004065
64	1437751_at	1.87139	6.09E-08	9.92E-06	Ppargc1a	peroxisome proliferative activated receptor, gamma, coactivator 1 alpha	5	AV337619
65	1456395_at	1.85383	1.17E-08	3.04E-06	Ppargc1a	peroxisome proliferative activated receptor, gamma, coactivator 1 alpha	5	BM120569
66	1434292_at	1.85189	9.75E-08	1.41E-05	Snhg11	small nucleolar RNA host gene 11 (non-protein coding)	2	BI731047
67	1460336_at	1.84569	4.48E-08	7.96E-06	Ppargc1a	peroxisome proliferative activated receptor, gamma, coactivator 1 alpha	5	BB745167
68	1421041_s_at	1.82573	4.29E-07	3.69E-05				NM_008182
69	1436853_a_at	1.81712	6.28E-05	0.00125154	Snca	synuclein, alpha	6	AI324124

70	1416432_at	1.81024	0.000284	0.0036871	Pfkfb3	6-phosphofructo-2-kinase/fructose-2,6-biphosphatase 3	2	NM_133232
71	1426980_s_at	1.79789	1.72E-10	1.74E-07	E130012A19Rik	RIKEN cDNA E130012A19 gene	11	BC006054
72	1443870_at	1.79035	1.27E-08	3.21E-06	Abcc4	ATP-binding cassette, sub-family C (CFTR/MRP), member 4	14	BB291885
73	1450970_at	1.79022	2.61E-08	5.51E-06	Got1	glutamate oxaloacetate transaminase 1, soluble	19	AA792094
74	1416411_at	1.78582	1.70E-10	1.74E-07	Gstm2	glutathione S-transferase, mu 2	3	NM_008183
75	1439617_s_at	1.76601	5.04E-06	0.000211858	Pck1	phosphoenolpyruvate carboxykinase 1, cytosolic	2	AI265463
76	1418780_at	1.76264	9.91E-08	1.42E-05	Cyp39a1	cytochrome P450, family 39, subfamily a, polypeptide 1	17	NM_018887
77	1429206_at	1.75531	1.11E-07	1.49E-05	Rhobtb1	Rho-related BTB domain containing 1	10	AK014194
78	1440084_at	1.74163	1.74E-05	0.000492191				AV380966
79	1452416_at	1.74109	6.54E-08	1.05E-05	Il6ra	interleukin 6 receptor, alpha	3	X53802
80	1436504_x_at	1.73114	7.06E-08	1.09E-05	Apoa4	apolipoprotein A-IV	9	AV027367
81	1422217_a_at	1.68022	1.80E-05	0.000503219	Cyp1a1	cytochrome P450, family 1, subfamily a, polypeptide 1	9	NM_009992
82	1458442_at	1.67999	0.00175723	0.0139958	AI132709	expressed sequence AI132709	7	AI266897
83	1428512_at	1.66989	3.53E-05	0.000827142	Bhlhb9	basic helix-loop-helix domain containing, class B9	X	AK012577
84	1436538_at	1.66965	0.00166756	0.0134757	Ankrd37	ankyrin repeat domain 37	8	AV084342
85	1434099_at	1.65966	9.59E-08	1.39E-05	Ppargc1a	peroxisome proliferative activated receptor, gamma, coactivator 1 alpha	5	BB752393
86	1429639_at	1.64707	3.90E-05	0.000880806	Gpcpd1	glycerophosphocholine phosphodiesterase GDE1 homolog (S. cerevisiae)	2	AK009137
87	1449525_at	1.64667	2.36E-08	5.24E-06	Fmo3	flavin containing monooxygenase 3	1	NM_008030
88	1423627_at	1.6453	6.53E-10	4.21E-07	Nqo1	NAD(P)H dehydrogenase, quinone 1	8	AV158882
89	1424683_at	1.63015	1.53E-09	6.90E-07	Fam134b	family with sequence similarity 134, member B	15	BC019494
90	1441971_at	1.62955	1.54E-05	0.000449462				AW543723
91	1450788_at	1.62411	0.00931516	0.0469457	Saa1	serum amyloid A 1	7	NM_009117
92	1449498_at	1.62282	1.14E-05	0.00037095	Marco	macrophage receptor with collagenous structure	1	NM_010766
93	1418595_at	1.62005	7.95E-06	0.000290898	Plin4	perilipin 4	17	NM_020568
94	1426850_a_at	1.61259	1.14E-06	7.52E-05	Map2k6	mitogen-activated protein kinase kinase 6	11	BB261602
95	1416332_at	1.60988	2.85E-06	0.000141625	Cirbp	cold inducible RNA binding protein	10	NM_007705
96	1437953_at	1.60608	1.67E-05	0.000477846	Gpcpd1	glycerophosphocholine phosphodiesterase GDE1 homolog (S. cerevisiae)	2	BM246706
97	1428352_at	1.60558	0.000503416	0.00557065	Arrdc2	arrestin domain containing 2	8	AW542672
98	1434100_x_at	1.60175	1.09E-07	1.48E-05	Ppargc1a	peroxisome proliferative activated receptor, gamma, coactivator 1 alpha	5	BB752393
99	1419075_s_at	1.59708	0.0137577	0.0620865				NM_011314
100	1440325_at	1.59699	0.00039847	0.00470615				AV332226
101	1431213_a_at	1.58832	0.000285772	0.00370224				BG297038
102	1456960_at	1.5838	0.0123625	0.0574266				BB555069
103	1457438_at	1.567	2.94E-07	2.89E-05				BE630363
104	1455869_at	1.54825	0.000245394	0.00331066				BG862223
105	1416953_at	1.54136	1.59E-06	9.43E-05	Ctgf	connective tissue growth factor	10	NM_010217

106	1438313_at	1.5313	0.00075336	0.00748557				BB233366
107	1418493_a_at	1.52271	0.00012644	0.0020301	Snca	synuclein, alpha	6	NM_009221
108	1428926_at	1.51782	2.11E-11	4.43E-08	1110003O08Rik	RIKEN cDNA 1110003O08 gene	8	AK003388
109	1435459_at	1.51389	3.20E-05	0.0007706	Fmo2	flavin containing monooxygenase 2	1	BM936480
110	1456156_at	1.50843	6.10E-08	9.92E-06	Lepr	leptin receptor	4	BM124366
111	1454799_at	1.49193	9.02E-05	0.00159979	Agpat9	1-acylglycerol-3-phosphate O-acyltransferase 9	5	AV300264
112	1427537_at	1.48986	7.19E-07	5.45E-05	Eppk1	epiplakin 1	15	BC026387
113	1425281_a_at	1.48115	0.00121457	0.0106793	Tsc22d3	TSC22 domain family, member 3	X	AF201289
114	1422230_s_at	1.47736	5.39E-10	3.76E-07				NM_007812
115	1428650_at	1.47609	7.19E-11	9.57E-08	Tns1	tensin 1	1	AK003780
116	1436948_a_at	1.46775	6.32E-07	4.96E-05	Fam70a	family with sequence similarity 70, member A	X	BB520013
117	1459897_a_at	1.45271	1.13E-05	0.000366724	Sbsn	suprabasin	7	AI507307
118	1419647_a_at	1.44305	0.000256439	0.00342176	Ier3	immediate early response 3	17	NM_133662
119	1433816_at	1.44031	7.66E-06	0.000282498	Mcart1	mitochondrial carrier triple repeat 1	4	BQ031264
120	1449326_x_at	1.44005	0.0490335	0.152346	Saa2	serum amyloid A 2	7	NM_011314
121	1455002_at	1.43506	7.32E-08	1.11E-05	Ptp4a1	protein tyrosine phosphatase 4a1	1	AV331223
122	1417602_at	1.42933	7.78E-06	0.000285692	Per2	period homolog 2 (Drosophila)	1	AF035830
123	1419329_at	1.42749	1.73E-09	7.44E-07	Sorbs3	sorbin and SH3 domain containing 3	14	NM_011366
124	1419024_at	1.42248	1.81E-05	0.000506149	Ptp4a1	protein tyrosine phosphatase 4a1	1	BC003761
125	1427425_at	1.42059	2.96E-08	6.02E-06	9130208E07Rik	RIKEN cDNA 9130208E07 gene	4	BC026435
126	1451355_at	1.41651	8.88E-10	4.65E-07	Acer2	alkaline ceramidase 2	4	AF282864
127	1417042_at	1.41171	3.79E-09	1.37E-06	Slc37a4	solute carrier family 37 (glucose-6-phosphate transporter), member 4	9	NM_008063
128	1440840_at	1.41121	7.01E-08	1.09E-05	D630004K10Rik	RIKEN cDNA D630004K10 gene	10	BB335455
129	1416926_at	1.4101	0.000259761	0.00345037	Trp53inp1	transformation related protein 53 inducible nuclear protein 1	4	AW495711
130	1419582_at	1.4005	8.49E-09	2.46E-06	Cyp2c55	cytochrome P450, family 2, subfamily c, polypeptide 55	19	NM_028089
131	1429050_at	1.40022	2.49E-08	5.37E-06	Chic2	cysteine-rich hydrophobic domain 2	5	AK015681
132	1448950_at	1.39128	1.77E-05	0.000497803	Il1r1	interleukin 1 receptor, type I	1	NM_008362
133	1423891_at	1.38748	4.82E-05	0.00102418	Gstt3	glutathione S-transferase, theta 3	10	BC003903
134	1445574_at	1.38493	4.47E-05	0.000975767				BG067678
135	1423439_at	1.38431	5.78E-06	0.000233975	Pck1	phosphoenolpyruvate carboxykinase 1, cytosolic	2	AW106963
136	1453851_a_at	1.37658	0.0100545	0.0496727	Gadd45g	growth arrest and DNA-damage-inducible 45 gamma	13	AK007410
137	1439293_at	1.36318	0.00114944	0.0102843	BC031353	cDNA sequence BC031353	9	BB369212
138	1452135_at	1.36202	3.41E-07	3.19E-05	Gpx6	glutathione peroxidase 6	13	AV001252
139	1422905_s_at	1.34691	1.71E-05	0.000485401	Fmo2	flavin containing monooxygenase 2	1	NM_018881
140	1426599_a_at	1.34454	0.000122087	0.00198634	Slc2a1	solute carrier family 2 (facilitated glucose transporter), member 1	4	BM209618
141	1435697_a_at	1.34394	1.48E-07	1.81E-05	Cytip	cytohesin 1 interacting protein	2	BB503614
142	1449851_at	1.34183	2.76E-05	0.000687934	Per1	period homolog 1 (Drosophila)	11	AF022992
143	1453101_at	1.33928	1.01E-05	0.000342324	Klhl25	kelch-like 25 (Drosophila)	7	AK012967
144	1417130_s_at	1.33813	0.000779471	0.00765161	Angptl4	angiopoietin-like 4	17	NM_020581
145	1449007_at	1.33548	3.07E-09	1.18E-06				NM_009770

146	1421852_at	1.33546	0.000991741	0.00921954	Kcnk5	potassium channel, subfamily K, member 5	14	AF319542
147	1435666_at	1.32614	6.92E-10	4.32E-07	Mast3	microtubule associated serine/threonine kinase 3	8	AW553439
148	1424629_at	1.31389	3.55E-07	3.26E-05	Brca1	breast cancer 1	11	U31625
149	1434456_at	1.3114	3.88E-07	3.44E-05	Rundc3b	RUN domain containing 3B	5	BG075955
150	1436194_at	1.30914	2.12E-07	2.31E-05	Prelid2	PRELI domain containing 2	18	BE985366
151	1434773_a_at	1.30634	0.000320374	0.00402038	Slc2a1	solute carrier family 2 (facilitated glucose transporter), member 1	4	BM207588
152	1454712_at	1.29993	1.69E-05	0.000479895	Mcart1	mitochondrial carrier triple repeat 1	4	AW212577
153	1434502_x_at	1.29665	2.52E-05	0.000645383	Slc4a1	solute carrier family 4 (anion exchanger), member 1	11	BB448377
154	1429671_at	1.2962	0.00012536	0.00202052	Scand3	SCAN domain containing 3	5	AK010551
155	1451452_a_at	1.29039	3.81E-05	0.000866233	Rgs16	regulator of G-protein signaling 16	1	U72881
156	1449945_at	1.27738	9.80E-06	0.000335034	Ppargc1b	peroxisome proliferative activated receptor, gamma, coactivator 1 beta	18	NM_133249
157	1441915_s_at	1.27147	2.16E-05	0.000572773	Plin5	perilipin 5	17	BB717485
158	1427258_at	1.27035	5.23E-08	8.92E-06	Trim24	tripartite motif-containing 24	6	BB611004
159	1424638_at	1.26536	0.0499934	0.154415	Cdkn1a	cyclin-dependent kinase inhibitor 1A (P21)	17	AK007630
160	1433604_x_at	1.2623	2.33E-10	2.20E-07	Aldoa	aldolase A, fructose-bisphosphate	7	BG065457
161	1433610_at	1.26094	0.000646487	0.00668611	AA986860	expressed sequence AA986860	1	BB522283
162	1451206_s_at	1.26012	7.58E-07	5.68E-05	Cytip	cytohesin 1 interacting protein	2	BC007144
163	1417969_at	1.25644	5.72E-10	3.89E-07	Fbxo31	F-box protein 31	8	NM_133765
164	1427638_at	1.255	0.00034247	0.0042342	Zbtb16	zinc finger and BTB domain containing 16	9	Z47205
165	1420630_at	1.25458	1.63E-11	4.43E-08	8430419L09Rik	RIKEN cDNA 8430419L09 gene	6	NM_028982
166	1418488_s_at	1.25332	5.81E-07	4.65E-05	Ripk4	receptor-interacting serine-threonine kinase 4	16	AF302127
167	1424401_at	1.25294	2.81E-07	2.80E-05	Aldh1l1	aldehyde dehydrogenase 1 family, member L1	6	AK007822
168	1421040_a_at	1.25158	1.20E-06	7.79E-05	Gsta2	glutathione S-transferase, alpha 2 (Yc2)	9	NM_008182
169	1458701_at	1.25062	3.00E-05	0.000735947	Gpcpd1	glycerophosphocholine phosphodiesterase GDE1 homolog (S. cerevisiae)	2	BB550273
170	1460510_a_at	1.2399	1.63E-05	0.000468515	Coq10b	coenzyme Q10 homolog B (S. cerevisiae)	1	AK006551
171	1418099_at	1.2369	4.58E-05	0.000992955	Tnfrsf1b	tumor necrosis factor receptor superfamily, member 1b	4	M60469
172	1424943_at	1.23684	0.00102641	0.00946678	Cyp4a31	cytochrome P450, family 4, subfamily a, polypeptide 31	4	BC013476
173	1449824_at	1.23488	8.02E-07	5.95E-05	Prg4	proteoglycan 4 (megakaryocyte stimulating factor, articular superficial zone protein)	1	NM_021400
174	1436766_at	1.2324	0.00469294	0.0286963	Luc7l2	LUC7-like 2 (S. cerevisiae)	6	BB475271
175	1424937_at	1.23001	0.000160818	0.00242277	Plin5	perilipin 5	17	BC024138
176	1416250_at	1.22376	0.00787945	0.0416813	Btg2	B-cell translocation gene 2, anti-proliferative	1	NM_007570
177	1448021_at	1.22165	2.45E-05	0.000630588				AA266723
178	1420772_a_at	1.22037	0.00635195	0.0357657	Tsc22d3	TSC22 domain family, member 3	X	NM_010286
179	1436544_at	1.2069	1.89E-08	4.30E-06	Atp10d	ATPase, class V, type 10D	5	BB016769
180	1426663_s_at	1.20548	0.00718174	0.0391279	Slc45a3	solute carrier family 45, member 3	1	BC024519
181	1419283_s_at	1.19979	2.72E-10	2.30E-07	Tns1	tensin 1	1	NM_027884
182	1432517_a_at	1.19936	1.32E-05	0.000408203	Nnmt	nicotinamide N-methyltransferase	9	AK006371

183	1429809_at	1.19724	3.23E-08	6.27E-06	Tmtc2	transmembrane and tetratricopeptide repeat containing 2	10	AK018506
184	1438815_at	1.18828	4.71E-05	0.0010077				BB360457
185	1449565_at	1.18812	2.96E-05	0.000729109	Cyp2g1	cytochrome P450, family 2, subfamily g, polypeptide 1	7	NM_013809
186	1429273_at	1.18617	1.16E-06	7.61E-05	Bmper	BMP-binding endothelial regulator	9	AK014221
187	1434799_x_at	1.18441	4.77E-10	3.41E-07	Aldoa	aldolase A, fructose-bisphosphate	7	BG793658
188	1425824_a_at	1.17838	0.000275115	0.00360368	Pcsk4	proprotein convertase subtilisin/kexin type 4	10	D01093
189	1443960_at	1.17668	2.25E-10	2.20E-07				BQ175377
190	1424599_at	1.17563	1.02E-05	0.000343048	Fgl1	fibrinogen-like protein 1	8	BC021946
191	1434059_at	1.17406	2.79E-11	5.10E-08	B230312A22Rik	RIKEN cDNA B230312A22 gene	4	BB769694
192	1434856_at	1.17396	1.38E-06	8.62E-05	Ankrd44	ankyrin repeat domain 44	1	AV256780
193	1416921_x_at	1.16625	1.31E-09	6.05E-07	Aldoa	aldolase A, fructose-bisphosphate	7	NM_007438
194	1442039_at	1.1662	8.23E-05	0.00150673	Tox	thymocyte selection-associated high mobility group box	4	BF020502
195	1460406_at	1.16415	3.89E-09	1.37E-06	Pls1	plastin 1 (I-isoform)	9	BC026410
196	1421681_at	1.16306	0.001264	0.0110297	Nrg4	neuregulin 4	9	NM_032002
197	1451069_at	1.15681	0.00271231	0.0191463	Pim3	proviral integration site 3	15	BC017621
198	1425979_a_at	1.15603	2.13E-06	0.00011738	Fbf1	Fas (TNFRSF6) binding factor 1	11	AF241249
199	1442406_at	1.15381	3.11E-05	0.000752094	9230104K21Rik	RIKEN cDNA 9230104K21 gene	4	BE852666
200	1424609_a_at	1.15371	0.0038008	0.0246036				BM225255
201	1432543_a_at	1.15357	2.81E-06	0.000140579	Klf13	Kruppel-like factor 13	7	AK002926
202	1425627_x_at	1.14535	1.76E-11	4.43E-08	Gstm1	glutathione S-transferase, mu 1	3	J03952
203	1417714_x_at	1.14438	0.000391144	0.00464397				NM_008218
204	1434817_s_at	1.14124	3.97E-07	3.49E-05	Rprd2	regulation of nuclear pre-mRNA domain containing 2	3	BM206427
205	1460011_at	1.14112	6.15E-05	0.0012345	Cyp26b1	cytochrome P450, family 26, subfamily b, polypeptide 1	6	AW049789
206	1427331_at	1.13792	7.14E-09	2.18E-06	Adora1	adenosine A1 receptor	1	BB518868
207	1458599_at	1.13658	3.01E-05	0.00073595				BB009155
208	1424175_at	1.13558	1.57E-06	9.39E-05	Tef	thyrotroph embryonic factor	15	BC017689
209	1418492_at	1.13349	0.0243578	0.0932616	Grem2	gremlin 2 homolog, cysteine knot superfamily (Xenopus laevis)	1	NM_011825
210	1453303_at	1.13112	1.22E-06	7.87E-05				BI076733
211	1456563_at	1.12942	2.36E-07	2.51E-05	4933429F08Rik	RIKEN cDNA 4933429F08 gene	18	BB769119
212	1453675_at	1.12849	1.00E-08	2.75E-06	Slc16a10	solute carrier family 16 (monocarboxylic acid transporters), member 10	10	AK011813
213	1446068_at	1.12641	0.000681048	0.00692198	Adk	adenosine kinase	14	BB053697
214	1451804_a_at	1.12556	1.79E-06	0.000102369	Lrrc16a	leucine rich repeat containing 16A	13	BC012229
215	1457117_at	1.12434	1.62E-05	0.000466411	Nfe2l2	nuclear factor, erythroid derived 2, like 2	2	AV248273
216	1424951_at	1.12402	2.16E-06	0.000118108	Baiap2l1	BAI1-associated protein 2-like 1	5	BC015459
217	1429656_at	1.12023	3.04E-05	0.000740189	Rhobtb1	Rho-related BTB domain containing 1	10	BB041370
218	1416034_at	1.11647	0.00102146	0.00943896	Cd24a	CD24a antigen	10	NM_009846
219	1426942_at	1.1138	1.07E-06	7.26E-05	Aim1	absent in melanoma 1	10	BM233292
220	1456405_at	1.1136	4.50E-05	0.000978478	Dido1	death inducer-obliterator 1	2	BG063067

221	1419393_at	1.10512	7.07E-10	4.32E-07	Abcg5	ATP-binding cassette, sub-family G (WHITE), member 5	17	NM_031884
222	1451814_a_at	1.10445	3.02E-09	1.18E-06	Htatip2	HIV-1 tat interactive protein 2, homolog (human)	7	AF061972
223	1427540_at	1.10263	7.75E-05	0.00143969	Zwint	ZW10 interactor	10	BC013559
224	1424607_a_at	1.10141	0.00266484	0.0189068				BM225255
225	1424050_s_at	1.10102	4.18E-05	0.000927633	Fgfr1	fibroblast growth factor receptor 1	8	M33760
226	1425626_at	1.10097	2.51E-11	4.91E-08	Gstm1	glutathione S-transferase, mu 1	3	J03952
227	1439375_x_at	1.10087	8.61E-10	4.64E-07				AV030922
228	1451263_a_at	1.09856	8.05E-07	5.96E-05	Fabp4	fatty acid binding protein 4, adipocyte	3	BC002148
229	1441042_at	1.0947	4.39E-09	1.51E-06	Fgf1	fibroblast growth factor 1	18	BE688115
230	1416464_at	1.0824	3.37E-05	0.000799768	Slc4a1	solute carrier family 4 (anion exchanger), member 1	11	NM_011403
231	1426858_at	1.08191	0.0135943	0.0615007				BB253137
232	1418249_at	1.07913	1.56E-10	1.69E-07	Crcp	calcitonin gene-related peptide-receptor component protein	5	NM_007761
233	1417023_a_at	1.0723	3.39E-06	0.000159398	Fabp4	fatty acid binding protein 4, adipocyte	3	NM_024406
234	1437247_at	1.06956	0.000163626	0.00244871				BM245170
235	1425921_a_at	1.06851	0.000272059	0.00356843	1810055G02Rik	RIKEN cDNA 1810055G02 gene	19	BC019471
236	1444226_at	1.0681	1.03E-06	7.10E-05	Foxo3	forkhead box O3	10	W07885
237	1448265_x_at	1.06427	6.78E-06	0.000261031	Mpzl2	myelin protein zero-like 2	9	BC015076
238	1431742_at	1.06127	0.00047495	0.00532402	1810053B23Rik	RIKEN cDNA 1810053B23 gene	16	AK007854
239	1435448_at	1.06076	1.57E-07	1.88E-05	Bcl2l11	BCL2-like 11 (apoptosis facilitator)	2	BM120925
240	1424977_at	1.05949	8.60E-07	6.24E-05	Lrrc67	leucine rich repeat containing 67	1	BC022722
241	1416773_at	1.05865	0.000146691	0.00226229	Wee1	WEE 1 homolog 1 (S. pombe)	7	NM_009516
242	1440443_at	1.05315	0.000205332	0.00290257	E030016H06Rik	RIKEN cDNA E030016H06 gene	2	BB531351
243	1426600_at	1.0504	0.000239496	0.00325713	Slc2a1	solute carrier family 2 (facilitated glucose transporter), member 1	4	BM209618
244	1436590_at	1.04631	0.0138275	0.0623195	Ppp1r3b	protein phosphatase 1, regulatory (inhibitor) subunit 3B	8	BG071940
245	1455345_at	1.04372	2.18E-05	0.000576521	Phf15	PHD finger protein 15	11	BI663145
246	1448330_at	1.04092	2.18E-12	9.13E-09	Gstm1	glutathione S-transferase, mu 1	3	NM_010358
247	1433757_a_at	1.03951	5.60E-05	0.00114854	Nisch	nischarin	14	BB025231
248	1460196_at	1.03848	2.47E-06	0.000128058	Cbr1	carbonyl reductase 1	16	NM_007620
249	1437100_x_at	1.03754	0.00379066	0.0245522	Pim3	proviral integration site 3	15	BB206220
250	1435483_x_at	1.03657	3.45E-05	0.000813381	Slc25a32	solute carrier family 25, member 32	15	AI662800
251	1431339_a_at	1.03517	6.12E-07	4.86E-05	Efhd2	EF hand domain containing 2	4	AK007560
252	1439830_at	1.03457	6.66E-09	2.07E-06	Map3k5	mitogen-activated protein kinase kinase kinase 5	10	AV377656
253	1427866_x_at	1.03426	0.000772769	0.00760365				AF071431
254	1449322_at	1.03392	3.89E-09	1.37E-06				BC003761
255	1434153_at	1.03354	0.000231641	0.00317369	Shb	src homology 2 domain-containing transforming protein B	4	BI408715
256	1454078_a_at	1.03329	5.51E-06	0.000225778	Gal3st1	galactose-3-O-sulfotransferase 1	11	AK002510
257	1425150_at	1.02869	0.0210283	0.0843032	Acnat2	acyl-coenzyme A amino acid N-acyltransferase 2	4	BC010829
258	1439630_x_at	1.02828	3.22E-07	3.07E-05	Sbsn	suprabasin	7	AI844734

259	1419758_at	1.02692	0.00194377	0.0151198	Abcb1a	ATP-binding cassette, sub-family B (MDR/TAP), member 1A	5	M30697
260	1460672_at	1.0257	3.55E-07	3.26E-05	2410002F23Rik	RIKEN cDNA 2410002F23 gene	7	BC016099
261	1422670_at	1.02382	2.75E-10	2.30E-07	Rnd2	Rho family GTPase 2	11	NM_009708
262	1460259_s_at	1.02365	7.38E-06	0.000275414				AF108501
263	1428143_a_at	1.02135	2.98E-08	6.02E-06	Pnpla2	patatin-like phospholipase domain containing 2	7	AK003207
264	1422570_at	1.01773	8.23E-06	0.000297865	Yy1	YY1 transcription factor	12	BI665246
265	1443673_x_at	1.01632	0.0017094	0.0136967				BB710847
266	1435860_at	1.01186	1.58E-06	9.42E-05	Slc5a6	solute carrier family 5 (sodium-dependent vitamin transporter), member 6	5	BF450030
267	1448529_at	1.01055	0.00676752	0.0375003	Thbd	thrombomodulin	2	NM_009378
268	1416258_at	1.00416	1.77E-07	2.00E-05	Tk1	thymidine kinase 1	11	NM_009387
269	1431334_a_at	1.00153	4.67E-07	3.93E-05	4933433P14Rik	RIKEN cDNA 4933433P14 gene	12	AK017049
270	1457123_at	1.00041	0.00829539	0.0432358	Nrg4	neuregulin 4	9	BB219343
271	1418322_at	-1.0001	6.93E-05	0.00134376	Crem	cAMP responsive element modulator	18	AI467599
272	1417963_at	-1.00075	0.000401977	0.0047387	Pltp	phospholipid transfer protein	2	NM_011125
273	1419123_a_at	-1.00127	9.84E-05	0.00170288	Pdgfc	platelet-derived growth factor, C polypeptide	3	NM_019971
274	1440226_at	-1.00668	8.09E-07	5.97E-05	Zfp760	zinc finger protein 760	17	BB088782
275	1428083_at	-1.00697	0.00649208	0.0363244	Neat1	nuclear paraspeckle assembly transcript 1 (non-protein coding)	19	AK018202
276	1439831_at	-1.00915	1.21E-08	3.09E-06				AW111920
277	1435775_at	-1.01008	4.05E-06	0.000181828	Clock	circadian locomoter output cycles kaput	5	BQ173970
278	1437939_s_at	-1.0103	0.000214367	0.00299399	Ctsc	cathepsin C	7	BM237633
279	1451485_at	-1.01483	8.58E-06	0.000307296	Luc7l3	LUC7-like 3 (S. cerevisiae)	11	AW536179
280	1425241_a_at	-1.01557	0.000972505	0.00909615	Wsb1	WD repeat and SOCS box-containing 1	11	BC019601
281	1422537_a_at	-1.01718	0.00467967	0.028645	Id2	inhibitor of DNA binding 2	12	NM_010496
282	1434129_s_at	-1.0178	0.000340807	0.00422077	Lhfpl2	lipoma HMGIC fusion partner-like 2	13	BG917242
283	1451730_at	-1.02189	3.13E-06	0.00015139	Zfp62	zinc finger protein 62	11	BC022935
284	1423797_at	-1.02266	0.0133131	0.0606402	Aacs	acetoacetyl-CoA synthetase	5	BC026817
285	1455182_at	-1.02326	5.27E-05	0.00109321	Kif1b	kinesin family member 1B	4	AV104668
286	1423569_at	-1.02359	3.08E-06	0.000150088	Gatm	glycine amidinotransferase (L-arginine:glycine amidinotransferase)	2	AW108522
287	1455025_at	-1.02775	3.86E-06	0.0001746	Paqr9	progestin and adipoQ receptor family member IX	9	AV103696
288	1448080_at	-1.03073	0.000259275	0.00344624				AI256288
289	1448183_a_at	-1.04068	4.28E-06	0.00018962	Hif1a	hypoxia inducible factor 1, alpha subunit	12	BB269715
290	1424022_at	-1.04158	0.00355198	0.0234257	Osgin1	oxidative stress induced growth inhibitor 1	8	BC022135
291	1417792_at	-1.04447	3.64E-06	0.000167038	Zfml	zinc finger, matrin-like	6	BM238431
292	1437773_x_at	-1.04661	7.39E-08	1.12E-05				BB476615
293	1435194_at	-1.0501	3.08E-07	2.98E-05	Hspa4	heat shock protein 4	11	AW909503
294	1455961_at	-1.05147	4.84E-06	0.000205897				AV174022
295	1436550_at	-1.05163	0.000153154	0.00234101	Fbxo30	F-box protein 30	10	BB706685
296	1422533_at	-1.05378	0.00147648	0.012354	Cyp51	cytochrome P450, family 51	5	NM_020010
297	1424375_s_at	-1.05455	3.81E-05	0.000866233	Gimap4	GTPase, IMAP family member 4	6	BC005577
298	1438596_at	-1.05652	0.000557487	0.00601897				AW114007

299	1450988_at	-1.05888	8.05E-05	0.00148019	Lgr5	leucine rich repeat containing G protein coupled receptor 5	10	BB751088
300	1436186_at	-1.06	0.0153597	0.0671945	E2f8	E2F transcription factor 8	7	BM247465
301	1428230_at	-1.06403	2.04E-05	0.000553679	Prkd3	protein kinase D3	17	BF160591
302	1452338_s_at	-1.067	1.43E-06	8.85E-05	Itsn1	intersectin 1 (SH3 domain protein 1A)	16	AA172344
303	1455037_at	-1.07071	1.35E-06	8.51E-05	Plxna2	plexin A2	1	BB002869
304	1416403_at	-1.07115	1.04E-07	1.46E-05	Abcb10	ATP-binding cassette, sub-family B (MDR/TAP), member 10	8	AV382118
305	1420886_a_at	-1.07323	2.45E-07	2.56E-05	Xbp1	X-box binding protein 1	11	NM_013842
306	1443783_x_at	-1.07588	0.00787792	0.0416813	H2-Aa	histocompatibility 2, class II antigen A, alpha	17	AV086906
307	1452349_x_at	-1.07764	2.43E-06	0.000127348				AI481797
308	1425206_a_at	-1.07948	6.68E-07	5.19E-05	Ube3a	ubiquitin protein ligase E3A	7	BB224620
309	1456328_at	-1.08006	5.71E-05	0.00116521	Bank1	B-cell scaffold protein with ankyrin repeats 1	3	AI451642
310	1423078_a_at	-1.08079	0.000673675	0.00685656	Sc4mol	sterol-C4-methyl oxidase-like	8	AK005441
311	1448944_at	-1.08196	2.59E-06	0.00013215	Nrp1	neuropilin 1	8	AK011144
312	1425028_a_at	-1.08286	0.000392275	0.00465174	Tpm2	tropomyosin 2, beta	4	BC024358
313	1436169_at	-1.08392	1.58E-08	3.75E-06	C730029A08Rik	RIKEN cDNA C730029A08 gene	9	BB428892
314	1440890_a_at	-1.084	9.33E-06	0.000321936	Zfp809	zinc finger protein 809	9	BQ176399
315	1425343_at	-1.08497	1.76E-05	0.000497353	Hdhd3	haloacid dehalogenase-like hydrolase domain containing 3	4	BC003491
316	1433901_at	-1.0864	7.92E-05	0.00146213	Caprin1	cell cycle associated protein 1	2	AV301998
317	1450743_s_at	-1.08681	1.27E-05	0.000396326	Syncrip	synaptotagmin binding, cytoplasmic RNA interacting protein	9	BG920261
318	1424303_at	-1.08763	1.88E-05	0.00052002	Depdc7	DEP domain containing 7	2	BC013499
319	1426278_at	-1.08831	0.000121836	0.00198392	Ifi27l2a	interferon, alpha-inducible protein 27 like 2A	12	AY090098
320	1435692_at	-1.08881	1.13E-08	2.96E-06	Kctd21	potassium channel tetramerisation domain containing 21	7	BQ176086
321	1451721_a_at	-1.08919	0.00224645	0.0167325	H2-Ab1	histocompatibility 2, class II antigen A, beta 1	17	M15848
322	1433623_at	-1.09842	3.13E-11	5.10E-08	Zfp367	zinc finger protein 367	13	BE629588
323	1460256_at	-1.10041	2.27E-06	0.000121799	Car3	carbonic anhydrase 3	3	NM_007606
324	1430893_at	-1.10151	2.27E-06	0.000121799	Mup10	major urinary protein 10	4	AK011413
325	1457883_at	-1.1059	3.54E-06	0.000164439				BB225963
326	1442082_at	-1.10715	5.84E-07	4.66E-05	C3ar1	complement component 3a receptor 1	6	BB333624
327	1459141_at	-1.10729	1.97E-06	0.000110259	1810008I18Rik	RIKEN cDNA 1810008I18 gene	7	BB667838
328	1448754_at	-1.11641	0.00045235	0.00515349	Rbp1	retinol binding protein 1, cellular	9	NM_011254
329	1449931_at	-1.11852	1.15E-05	0.000372975	Cpeb4	cytoplasmic polyadenylation element binding protein 4	11	NM_026252
330	1451753_at	-1.12021	4.49E-09	1.53E-06	Plxna2	plexin A2	1	D86949
331	1422769_at	-1.12956	9.56E-05	0.00166512	Syncrip	synaptotagmin binding, cytoplasmic RNA interacting protein	9	BG920261
332	1426464_at	-1.13123	0.00107735	0.00980696	Nr1d1	nuclear receptor subfamily 1, group D, member 1	11	W13191
333	1429006_s_at	-1.13198	3.39E-06	0.000159398	2610110G12Rik	RIKEN cDNA 2610110G12 gene	17	AK011838
334	1439300_at	-1.13275	0.000203872	0.00288872	Chic1	cysteine-rich hydrophobic domain 1	X	BG065782
335	1437864_at	-1.13314	3.68E-06	0.000168105	Adipor2	adiponectin receptor 2	6	BE632137
336	1425993_a_at	-1.13642	0.00226228	0.0168204	Hsph1	heat shock 105kDa/110kDa protein 1	5	D67017

337	1443505_at	-1.13676	3.27E-06	0.000156079				BE651535
338	1452374_at	-1.14305	2.46E-08	5.37E-06	Zfp322a	zinc finger protein 322A	13	BB315154
339	1437085_at	-1.15306	0.00011634	0.00191892	D630039A03Rik	RIKEN cDNA D630039A03 gene	4	AV370040
340	1431900_a_at	-1.16145	3.16E-06	0.000152167	Foxa3	forkhead box A3	7	AK019022
341	1454617_at	-1.1616	0.0209753	0.084275	Arrdc3	arrestin domain containing 3	13	BG072824
342	1428229_at	-1.17543	2.28E-06	0.000122311	Prkd3	protein kinase D3	17	BF160591
343	1429772_at	-1.17811	7.20E-08	1.10E-05	Plxna2	plexin A2	1	BB085537
344	1453552_at	-1.17887	2.17E-05	0.00057625	2310014F07Rik	RIKEN cDNA 2310014F07 gene	9	AK009336
345	1436387_at	-1.17897	3.02E-05	0.000738195	C330006P03Rik	RIKEN cDNA C330006P03 gene	13	BB398124
346	1442367_at	-1.18929	1.17E-05	0.000375708	Atp11c	ATPase, class VI, type 11C	X	BB184010
347	1439837_at	-1.19241	3.21E-08	6.27E-06	Gigyf2	GRB10 interacting GYF protein 2	1	BE136147
348	1449109_at	-1.19402	0.0225243	0.0884059	Socs2	suppressor of cytokine signaling 2	10	NM_007706
349	1429184_at	-1.19557	1.52E-05	0.000444652	Gvin1	GTPase, very large interferon inducible 1	7	BM243571
350	1453286_at	-1.19932	1.86E-07	2.07E-05	Plxna2	plexin A2	1	BB085537
351	1433443_a_at	-1.20603	0.000208645	0.00293505	Hmgcs1	3-hydroxy-3-methylglutaryl-Coenzyme A synthase 1	13	BB705380
352	1423319_at	-1.20787	0.00984526	0.0488711	Hhex	hematopoietically expressed homeobox	19	AK014111
353	1433444_at	-1.2089	0.000161919	0.0024331	Hmgcs1	3-hydroxy-3-methylglutaryl-Coenzyme A synthase 1	13	BB705380
354	1444512_at	-1.21625	0.000114292	0.00189423	Arhgap29	Rho GTPase activating protein 29	3	AI643890
355	1418804_at	-1.21902	5.68E-08	9.46E-06	Sucnr1	succinate receptor 1	3	NM_032400
356	1437581_at	-1.22286	6.58E-05	0.00129489	Zfp800	zinc finger protein 800	6	AW824355
357	1418930_at	-1.22642	1.54E-07	1.86E-05	Cxcl10	chemokine (C-X-C motif) ligand 10	5	NM_021274
358	1416022_at	-1.22712	0.00699154	0.0383827	Fabp5	fatty acid binding protein 5, epidermal	3	BC002008
359	1423045_at	-1.23045	5.14E-08	8.81E-06	Ncbp2	nuclear cap binding protein subunit 2	16	BE285362
360	1453282_at	-1.2355	1.08E-09	5.28E-07	Cxadr	coxsackie virus and adenovirus receptor	16	BE824924
361	1423566_a_at	-1.236	0.00119481	0.0105804	Hsph1	heat shock 105kDa/110kDa protein 1	5	BI499717
362	1416149_at	-1.23634	3.62E-05	0.000839292	Olig1	oligodendrocyte transcription factor 1	16	AB038696
363	1430401_at	-1.24045	4.06E-06	0.000181881	3110045C21Rik	RIKEN cDNA 3110045C21 gene	1	AK014177
364	1456898_at	-1.24294	2.12E-11	4.43E-08				AI426862
365	1456974_at	-1.24616	8.02E-06	0.000292593	Onecut1	one cut domain, family member 1	9	BG067274
366	1424727_at	-1.24666	4.85E-05	0.00102603	Ccr5	chemokine (C-C motif) receptor 5	9	D83648
367	1450783_at	-1.24811	3.51E-05	0.000823299	Ifit1	interferon-induced protein with tetratricopeptide repeats 1	19	NM_008331
368	1422144_at	-1.2492	9.10E-05	0.00160675	Inhbe	inhibin beta E	10	BC010404
369	1416021_a_at	-1.2502	0.00440181	0.0273458				BC002008
370	1439445_x_at	-1.25157	7.05E-08	1.09E-05	Acly	ATP citrate lyase	11	AV347837
371	1447543_at	-1.25498	4.28E-06	0.00018962	Wdfy1	WD repeat and FYVE domain containing 1	1	BB225041
372	1415965_at	-1.257	8.68E-05	0.00155977	Scd1	stearoyl-Coenzyme A desaturase 1	19	NM_009127
373	1417793_at	-1.26403	6.82E-06	0.000261031	Irgm2	immunity-related GTPase family M member 2	11	NM_019440
374	1426645_at	-1.26548	0.000657381	0.00675406	Hsp90aa1	heat shock protein 90, alpha (cytosolic), class A member 1	12	AU079047
375	1433445_x_at	-1.26907	8.37E-05	0.00152306	Hmgcs1	3-hydroxy-3-methylglutaryl-Coenzyme A synthase 1	13	BB705380
376	1427347_s_at	-1.26912	0.00631388	0.0356205	Tubb2a	tubulin, beta 2A	13	BC003475

377	1433575_at	-1.2707	0.000391785	0.00464969	Sox4	SRY-box containing gene 4	13	BG083485
378	1431302_a_at	-1.27186	1.69E-08	3.91E-06	Nudt7	nudix (nucleoside diphosphate linked moiety X)-type motif 7	8	AK011172
379	1460694_s_at	-1.27416	8.71E-10	4.64E-07	Svil	supervillin	18	BM203457
380	1427356_at	-1.27567	2.50E-06	0.000129245	Fam89a	family with sequence similarity 89, member A	8	BC023460
381	1417292_at	-1.2787	3.38E-08	6.47E-06	Ifi47	interferon gamma inducible protein 47	11	NM_008330
382	1439566_at	-1.27918	0.000152693	0.00233579	Gprin3	GPRIN family member 3	6	BB245373
383	1424842_a_at	-1.28355	4.85E-06	0.000205897	Arhgap24	Rho GTPase activating protein 24	5	BC025502
384	1420549_at	-1.28593	0.000111628	0.0018659	Gbp1	guanylate binding protein 1	3	NM_010259
385	1450090_at	-1.29128	9.12E-07	6.49E-05	Zfp101	zinc finger protein 101	17	NM_009542
386	1427838_at	-1.29561	0.00102563	0.0094625	Tubb2a	tubulin, beta 2A	13	M28739
387	1437119_at	-1.30192	9.31E-07	6.56E-05	Ern1	endoplasmic reticulum (ER) to nucleus signalling 1	11	BG075179
388	1436576_at	-1.30662	1.65E-05	0.000474053	Fam26f	family with sequence similarity 26, member F	10	BB239429
389	1422735_at	-1.31353	0.00357989	0.023562	Foxq1	forkhead box Q1	13	NM_008239
390	1450484_a_at	-1.31364	2.33E-08	5.21E-06	Cmpk2	cytidine monophosphate (UMP-CMP) kinase 2, mitochondrial	12	AK004595
391	1416840_at	-1.31373	0.00159641	0.0130665	Mid1ip1	Mid1 interacting protein 1 (gastrulation specific G12-like (zebrafish))	X	NM_026524
392	1418507_s_at	-1.32192	0.0229558	0.089484	Socs2	suppressor of cytokine signaling 2	10	NM_007706
393	1448663_s_at	-1.32257	0.00015186	0.00232852	Mvd	mevalonate (diphospho) decarboxylase	8	NM_138656
394	1453500_at	-1.32528	2.21E-09	9.10E-07	Cyp2u1	cytochrome P450, family 2, subfamily u, polypeptide 1	3	AK018458
395	1434135_at	-1.33059	9.04E-10	4.65E-07	B3galnt2	UDP-GalNAc:betaGlcNAc beta 1,3-galactosaminyltransferase, polypeptide 2	13	BB107552
396	1429954_at	-1.3341	2.31E-06	0.000123315	Clec4a3	C-type lectin domain family 4, member a3	6	AK014135
397	1416630_at	-1.33979	0.00183703	0.014509	Id3	inhibitor of DNA binding 3	4	NM_008321
398	1419547_at	-1.3455	2.10E-12	9.13E-09	Fahd1	fumarylacetoacetate hydrolase domain containing 1	17	BC026949
399	1438009_at	-1.34868	0.000446293	0.0051106				W91024
400	1449514_at	-1.36235	1.62E-08	3.79E-06	Grk5	G protein-coupled receptor kinase 5	19	BC019379
401	1418835_at	-1.36358	0.00904333	0.0460068	Phlda1	pleckstrin homology-like domain, family A, member 1	10	NM_009344
402	1451418_a_at	-1.36857	1.00E-06	6.95E-05	Spsb4	splA/ryanodine receptor domain and SOCS box containing 4	9	BC023083
403	1448724_at	-1.37539	0.00759107	0.0406049	Cish	cytokine inducible SH2-containing protein	9	NM_009895
404	1423046_s_at	-1.37545	1.35E-10	1.58E-07	Ncbp2	nuclear cap binding protein subunit 2	16	BE285362
405	1437756_at	-1.37679	5.11E-05	0.00106588	Gimap9	GTPase, IMAP family member 9	6	BF682515
406	1416833_at	-1.39098	9.94E-08	1.42E-05	Keg1	kidney expressed gene 1	19	NM_029550
407	1449844_at	-1.39135	8.55E-05	0.00154762	Slco1a1	solute carrier organic anion transporter family, member 1a1	6	AB031813
408	1449854_at	-1.39749	0.00323317	0.0218795	Nr0b2	nuclear receptor subfamily 0, group B, member 2	4	BC019540
409	1423418_at	-1.42692	0.000441149	0.00507083	Fdps	farnesyl diphosphate synthetase	3	BI247584
410	1420835_at	-1.43299	0.000105662	0.00179692	Slc25a30	solute carrier family 25, member 30	14	BB032012
411	1439377_x_at	-1.44668	1.49E-05	0.000441849	Cdc20	cell division cycle 20 homolog (S. cerevisiae)	4	BB041150

412	1425270_at	-1.45057	7.30E-05	0.00138678	Kif1b	kinesin family member 1B	4	BE199508
413	1450779_at	-1.45638	0.000625992	0.00651147	Fabp7	fatty acid binding protein 7, brain	10	NM_021272
414	1416039_x_at	-1.4584	0.000123656	0.00200407	Cyr61	cysteine rich protein 61	3	NM_010516
415	1427301_at	-1.46166	6.33E-07	4.96E-05	Cd48	CD48 antigen	1	BE634960
416	1424857_a_at	-1.47824	1.15E-07	1.54E-05	Trim34	tripartite motif-containing 34	7	AF220142
417	1452348_s_at	-1.48071	2.59E-06	0.00013215				AI481797
418	1418496_at	-1.48426	2.12E-06	0.00011738	Foxa1	forkhead box A1	12	NM_008259
419	1430834_at	-1.53288	0.000121819	0.00198392	Gprin3	GPRIN family member 3	6	BB359379
420	1450264_a_at	-1.53631	1.92E-05	0.000530618	Chka	choline kinase alpha	19	NM_013490
421	1415993_at	-1.53923	0.000706257	0.00711664	Sqle	squalene epoxidase	15	NM_009270
422	1438558_x_at	-1.54503	0.00537767	0.0317936	Foxq1	forkhead box Q1	13	AV009267
423	1433446_at	-1.56976	3.45E-06	0.000161148	Hmgcs1	3-hydroxy-3-methylglutaryl-Coenzyme A synthase 1	13	BB705380
424	1423571_at	-1.58054	1.33E-08	3.31E-06	S1pr1	sphingosine-1-phosphate receptor 1	3	BB133079
425	1418652_at	-1.60313	6.03E-05	0.00121853	Cxcl9	chemokine (C-X-C motif) ligand 9	5	NM_008599
426	1423804_a_at	-1.62327	0.000195558	0.00279797	Idi1	isopentenyl-diphosphate delta isomerase	13	BC004801
427	1424033_at	-1.63702	3.62E-06	0.000166498	Sfrs7	splicing factor, arginine/serine-rich 7	17	BC014857
428	1423397_at	-1.64102	0.000178864	0.00262448				AI118428
429	1448698_at	-1.64964	8.91E-11	1.14E-07	Ccnd1	cyclin D1	7	NM_007631
430	1438751_at	-1.654	9.20E-06	0.000319517	Slc30a10	solute carrier family 30, member 10	1	BB736474
431	1449009_at	-1.65632	1.13E-05	0.000366724				NM_011579
432	1433944_at	-1.6809	1.47E-07	1.81E-05	Hectd2	HECT domain containing 2	19	AV256030
433	1451122_at	-1.69138	5.37E-05	0.00110826	Idi1	isopentenyl-diphosphate delta isomerase	13	BC004801
434	1437453_s_at	-1.70953	1.24E-06	7.91E-05	Pcsk9	proprotein convertase subtilisin/kexin type 9	4	AV010795
435	1442537_at	-1.721	5.84E-10	3.89E-07				BB771206
436	1454842_a_at	-1.74703	9.19E-09	2.64E-06	B3galnt2	UDP-GalNAc:betaGlcNAc beta 1,3-galactosaminyltransferase, polypeptide 2	13	AI853240
437	1435462_at	-1.78992	1.30E-06	8.27E-05	Plcxd2	phosphatidylinositol-specific phospholipase C, X domain containing 2	16	BQ176176
438	1438676_at	-1.79767	5.56E-06	0.000227244	Mpa2l	macrophage activation 2 like	5	BM241485
439	1417419_at	-1.82335	5.52E-11	8.51E-08	Ccnd1	cyclin D1	7	NM_007631
440	1455324_at	-1.82846	2.80E-07	2.80E-05	Plcxd2	phosphatidylinositol-specific phospholipase C, X domain containing 2	16	BQ176176
441	1430785_at	-1.83813	3.11E-08	6.16E-06	Sdr9c7	4short chain dehydrogenase/reductase family 9C, member 7	10	BB150587
442	1420379_at	-1.84103	1.09E-06	7.30E-05	Slco1a1	solute carrier organic anion transporter family, member 1a1	6	AB031813
443	1447927_at	-1.86204	2.42E-07	2.55E-05	Mpa2l	macrophage activation 2 like	5	BG092512
444	1427513_at	-1.89121	4.73E-10	3.41E-07	BC024137	cDNA sequence BC024137	8	BI144810
445	1424709_at	-1.91773	2.82E-10	2.30E-07	Sc5d	sterol-C5-desaturase (fungal ERG3, delta-5-desaturase) homolog (S. cerevisae)	9	AB016248
446	1456074_at	-1.91916	1.17E-07	1.55E-05	Sdr9c7	4short chain dehydrogenase/reductase family 9C, member 7	10	BB143568
447	1430896_s_at	-1.93123	7.51E-09	2.25E-06	Nudt7	nudix (nucleoside diphosphate linked moiety X)-type motif 7	8	AK008824

448	1427422_at	-1.93675	0.00175004	0.0139498	Gm6484	predicted gene 6484	9	BM122014
449	1452318_a_at	-1.95598	0.00101704	0.00941592	Hspa1b	heat shock protein 1B	17	M12573
450	1420836_at	-1.971	9.08E-06	0.000318716	Slc25a30	solute carrier family 25, member 30	14	BB032012
451	1453588_at	-1.99988	0.00167797	0.0135299	Car3	carbonic anhydrase 3	3	BB213876
452	1437073_x_at	-2.004	1.02E-05	0.000344813				BB115446
453	1448986_x_at	-2.0871	6.52E-09	2.06E-06	Dnase2a	deoxyribonuclease II alpha	8	NM_010062
454	1421092_at	-2.08749	4.39E-05	0.00095845	Serpina12	serine (or cysteine) peptidase inhibitor, clade A (alpha-1 antiproteinase, antitrypsin), member 12	12	AK014346
455	1420722_at	-2.08772	1.06E-06	7.21E-05	Elovl3	elongation of very long chain fatty acids (FEN1/Elo2, SUR4/Elo3, yeast)-like 3	19	BC016468
456	1450018_s_at	-2.09056	2.73E-05	0.000684816	Slc25a30	solute carrier family 25, member 30	14	BB032012
457	1427127_x_at	-2.09413	0.000472756	0.00530755	Hspa1b	heat shock protein 1B	17	M12573
458	1433898_at	-2.10633	4.14E-05	0.0009228				AV000840
459	1450839_at	-2.16883	3.83E-06	0.000173563	D0H4S114	DNA segment, human D4S114	18	D45203
460	1427126_at	-2.218	0.000479138	0.00535649	Hspa1b	heat shock protein 1B	17	M12573
461	1417420_at	-2.23435	8.44E-14	1.04E-09	Ccnd1	cyclin D1	7	NM_007631
462	1420531_at	-2.25893	7.98E-06	0.000291427	Hsd3b5	hydroxy-delta-5-steroid dehydrogenase, 3 beta- and steroid delta-isomerase 5	3	NM_008295
463	1430584_s_at	-2.29317	0.000805274	0.00786538	Car3	carbonic anhydrase 3	3	BB213876
464	1417065_at	-2.29922	0.00027019	0.00355028	Egr1	early growth response 1	18	NM_007913
465	1427981_a_at	-2.37276	8.55E-08	1.25E-05	Csad	cysteine sulfinic acid decarboxylase	15	AY033912
466	1434520_at	-2.3861	4.30E-10	3.41E-07	Sc5d	sterol-C5-desaturase (fungal ERG3, delta-5-desaturase) homolog (S. cerevisae)	9	AU067703
467	1450252_at	-2.47701	0.00224798	0.0167396	Onecut1	one cut domain, family member 1	9	NM_008262
468	1431817_at	-2.50992	1.03E-09	5.21E-07	Adh6-ps1	alcohol dehydrogenase 6 (class V), pseudogene 1	3	AK004863
469	1444296_a_at	-2.67106	1.89E-07	2.09E-05	Serpina4-ps1	serine (or cysteine) peptidase inhibitor, clade A, member 4, pseudogene 1	12	BF383739
470	1444297_at	-2.72051	6.72E-07	5.21E-05	Serpina4-ps1	serine (or cysteine) peptidase inhibitor, clade A, member 4, pseudogene 1	12	BF383739
471	1436736_x_at	-2.89524	1.13E-07	1.51E-05	D0H4S114	DNA segment, human D4S114	18	BB369191
472	1421447_at	-2.9253	0.000910095	0.00864283				NM_008262
473	1448092_x_at	-3.26883	3.86E-08	7.21E-06	Serpina4-ps1	serine (or cysteine) peptidase inhibitor, clade A, member 4, pseudogene 1	12	AA267743

SHS^{4+1R} vs C

	Probeset_id	Log2Ratio.1	P.Value	Adj.P.Value	Symbol	Description	Chromosome	GenBank
1	1443147_at	4.13179	1.48E-07	7.31E-05				BB505010
2	1426037_a_at	4.10832	2.05E-06	0.000297843	Rgs16	regulator of G-protein signaling 16	1	U94828
3	1455265_a_at	3.28591	1.02E-06	0.000189984	Rgs16	regulator of G-protein signaling 16	1	BB100249
4	1417168_a_at	3.16386	6.68E-08	5.70E-05	Usp2	ubiquitin specific peptidase 2	9	AI553394
5	1422925_s_at	3.13776	3.55E-07	0.000109493	Acot3	acyl-CoA thioesterase 3	12	NM_134246
6	1417169_at	2.7911	1.38E-08	3.01E-05	Usp2	ubiquitin specific peptidase 2	9	AI553394
7	1418288_at	1.93146	0.000279407	0.00705792	Lpin1	lipin 1	12	NM_015763

8	1417904_at	1.90865	1.62E-07	7.65E-05	Dclre1a	DNA cross-link repair 1A, PSO2 homolog (S. cerevisiae)	19	AF241240
9	1435188_at	1.88137	3.13E-06	0.000377288	Gm129	predicted gene 129	3	BB407125
10	1426516_a_at	1.86847	0.000345911	0.00814782	Lpin1	lipin 1	12	AK014526
11	1458040_at	1.80034	3.43E-06	0.000403052				BM213832
12	1451452_a_at	1.78544	5.18E-07	0.000132036	Rgs16	regulator of G-protein signaling 16	1	U72881
13	1428223_at	1.74716	0.000484327	0.0101009	Mfsd2a	major facilitator superfamily domain containing 2A	4	AK006096
14	1425824_a_at	1.72075	3.02E-06	0.000371298	Pcsk4	proprotein convertase subtilisin/kexin type 4	10	D01093
15	1428923_at	1.7118	0.00189512	0.0250365	Ppp1r3g	protein phosphatase 1, regulatory (inhibitor) subunit 3G	13	AK005570
16	1418174_at	1.68441	0.0029	0.0338145	Dbp	D site albumin promoter binding protein	7	BC018323
17	1425837_a_at	1.65575	0.00353622	0.0386348				AF199491
18	1438211_s_at	1.65392	0.00363457	0.0393861	Dbp	D site albumin promoter binding protein	7	BB550183
19	1451548_at	1.6416	8.62E-05	0.00331895	Upp2	uridine phosphorylase 2	2	BC027189
20	1424969_s_at	1.62993	3.19E-05	0.00178615	Upp2	uridine phosphorylase 2	2	BC027189
21	1422557_s_at	1.62793	0.00480169	0.0468997	Mt1	metallothionein 1	8	NM_013602
22	1416432_at	1.60329	0.000909886	0.0155824	Pfkfb3	6-phosphofructo-2-kinase/fructose-2,6-biphosphatase 3	2	NM_133232
23	1451190_a_at	1.59469	1.02E-06	0.000189984	Sbk1	SH3-binding kinase 1	7	BC025837
24	1422257_s_at	1.58981	0.000763481	0.0139126	Cyp2b10	cytochrome P450, family 2, subfamily b, polypeptide 10	7	NM_009998
25	1425645_s_at	1.56264	0.000602441	0.0117763	Cyp2b10	cytochrome P450, family 2, subfamily b, polypeptide 10	7	AF128849
26	1416773_at	1.53407	1.47E-06	0.000236443	Wee1	WEE 1 homolog 1 (S. pombe)	7	NM_009516
27	1417602_at	1.50929	3.79E-06	0.000429376	Per2	period homolog 2 (Drosophila)	1	AF035830
28	1421087_at	1.47841	1.20E-06	0.000212469	Per3	period homolog 3 (Drosophila)	4	NM_011067
29	1449851_at	1.46472	9.24E-06	0.000784202	Per1	period homolog 1 (Drosophila)	11	AF022992
30	1416125_at	1.41848	1.76E-05	0.00122329	Fkbp5	FK506 binding protein 5	17	U16959
31	1438743_at	1.41796	0.0378923	0.176972	Cyp7a1	cytochrome P450, family 7, subfamily a, polypeptide 1	4	BB667338
32	1438431_at	1.39891	0.00261472	0.0313874	Abcd2	ATP-binding cassette, sub-family D (ALD), member 2	15	BB197269
33	1423978_at	1.39573	5.17E-07	0.000132036	Sbk1	SH3-binding kinase 1	7	BC025837
34	1428487_s_at	1.3825	1.37E-05	0.00102245	Coq10b	coenzyme Q10 homolog B (S. cerevisiae)	1	AK002294
35	1421681_at	1.37335	0.000262668	0.0067992	Nrg4	neuregulin 4	9	NM_032002
36	1421830_at	1.36272	7.76E-08	5.70E-05				NM_009647
37	1425646_at	1.35743	0.000129729	0.00426156	BC016495	cDNA sequence BC016495	19	BC016495
38	1456812_at	1.35119	6.01E-06	0.000580985	Abcd2	ATP-binding cassette, sub-family D (ALD), member 2	15	AW456685
39	1424175_at	1.34108	1.39E-07	7.03E-05	Tef	thyrotroph embryonic factor	15	BC017689
40	1451787_at	1.33535	0.000902184	0.015513	Cyp2b10	cytochrome P450, family 2, subfamily b, polypeptide 10	7	AF128849
41	1460510_a_at	1.33523	6.32E-06	0.00059463	Coq10b	coenzyme Q10 homolog B (S. cerevisiae)	1	AK006551
42	1419874_x_at	1.31674	0.0264209	0.141507	Zbtb16	zinc finger and BTB domain containing 16	9	AA419994

43	1442025_a_at	1.31194	0.0430913	0.191439				AI467657
44	1429206_at	1.291	8.53E-06	0.000742801	Rhobtb1	Rho-related BTB domain containing 1	10	AK014194
45	1416933_at	1.27923	5.27E-08	5.52E-05	Por	P450 (cytochrome) oxidoreductase	5	NM_008898
46	1431339_a_at	1.26585	2.91E-08	4.06E-05	Efhd2	EF hand domain containing 2	4	AK007560
47	1440840_at	1.25735	3.98E-07	0.00011675	D630004K10Rik	RIKEN cDNA D630004K10 gene	10	BB335455
48	1457123_at	1.24919	0.0015803	0.0222453	Nrg4	neuregulin 4	9	BB219343
49	1424815_at	1.24368	8.35E-07	0.000179812	Gys2	glycogen synthase 2	6	BC021322
50	1431806_at	1.23182	0.000129312	0.00425265	4931408D14Rik	RIKEN cDNA 4931408D14 gene	19	AK016444
51	1425895_a_at	1.21944	0.00571114	0.0524273	Id1	inhibitor of DNA binding 1	2	U43884
52	1449565_at	1.20857	2.40E-05	0.00153427	Cyp2g1	cytochrome P450, family 2, subfamily g, polypeptide 1	7	NM_013809
53	1435860_at	1.20395	1.25E-07	6.67E-05	Slc5a6	solute carrier family 5 (sodium-dependent vitamin transporter), member 6	5	BF450030
54	1450387_s_at	1.20374	1.34E-06	0.000224272				NM_009647
55	1459145_at	1.17624	3.22E-06	0.000383336	A930033H14Rik	RIKEN cDNA A930033H14 gene	10	BB483368
56	1448506_at	1.16525	1.40E-08	3.01E-05	Serpina6	serine (or cysteine) peptidase inhibitor, clade A, member 6	12	NM_007618
57	1421852_at	1.15865	0.0032186	0.0364136	Kcnk5	potassium channel, subfamily K, member 5	14	AF319542
58	1416029_at	1.15512	0.0248502	0.136003	Klf10	Kruppel-like factor 10	15	NM_013692
59	1453416_at	1.1488	3.51E-05	0.00190256	Gas2l3	growth arrest-specific 2 like 3	10	BE199211
60	1437478_s_at	1.13859	1.95E-09	1.84E-05	Efhd2	EF hand domain containing 2	4	AA409309
61	1429809_at	1.12689	8.29E-08	5.70E-05	Tmtc2	transmembrane and tetratricopeptide repeat containing 2	10	AK018506
62	1452416_at	1.12497	2.79E-05	0.00165706	Il6ra	interleukin 6 receptor, alpha	3	X53802
63	1452841_at	1.12269	3.59E-06	0.000417195	Pgm2l1	phosphoglucomutase 2-like 1	7	BG073164
64	1448568_a_at	1.11232	3.04E-05	0.00173341	Slc20a1	solute carrier family 20, member 1	2	NM_015747
65	1448978_at	1.10778	9.06E-08	5.70E-05	Ngef	neuronal guanine nucleotide exchange factor	1	NM_019867
66	1448162_at	1.10062	4.45E-05	0.00218559	Vcam1	vascular cell adhesion molecule 1	3	BB250384
67	1423797_at	1.09312	0.00881556	0.0700791	Aacs	acetoacetyl-CoA synthetase	5	BC026817
68	1432543_a_at	1.07722	6.99E-06	0.000642075	Klf13	Kruppel-like factor 13	7	AK002926
69	1434442_at	1.0752	3.18E-06	0.000379801	Stbd1	starch binding domain 1	5	BB667844
70	1460258_at	1.07517	5.70E-05	0.00254201	Lect1	leukocyte cell derived chemotaxin 1	14	NM_010701
71	1433858_at	1.07355	0.000950158	0.0160841	Lrrc28	leucine rich repeat containing 28	7	BB667092
72	1417761_at	1.07345	0.000143618	0.00454951	Apoa4	apolipoprotein A-IV	9	BC010769
73	1419758_at	1.06699	0.00140976	0.0206434	Abcb1a	ATP-binding cassette, sub-family B (MDR/TAP), member 1A	5	M30697
74	1448666_s_at	1.05889	0.000125375	0.00415582	Tob2	transducer of ERBB2, 2	15	AV174616
75	1449519_at	1.05401	0.0155885	0.1009	Gadd45a	growth arrest and DNA-damage-inducible 45 alpha	6	NM_007836
76	1426288_at	1.04805	2.32E-07	8.93E-05	Lrp4	low density lipoprotein receptor-related protein 4	2	AF247637
77	1435459_at	1.04439	0.00144658	0.0209954	Fmo2	flavin containing monooxygenase 2	1	BM936480
78	1450188_s_at	1.03825	0.0264348	0.141556	Lipg	lipase, endothelial	18	BC020991
79	1434437_x_at	1.027	0.00245673	0.0300281	Rrm2	ribonucleotide reductase M2	12	AV301324
80	1439189_at	1.0258	0.000180086	0.00531407	Fnip2	folliculin interacting protein 2	3	BB498793
81	1448902_at	1.02535	6.37E-05	0.00274851	Ttc23	tetratricopeptide repeat domain 23	7	NM_025905

82	1455665_at	1.02488	0.000366413	0.00851438				BB705689
83	1422077_at	1.00637	7.62E-06	0.000685167	Acot4	acyl-CoA thioesterase 4	12	AA571017
84	1422230_s_at	1.00571	2.56E-07	9.51E-05				NM_007812
85	1423214_at	-1.00129	9.15E-05	0.00344627	Plxnc1	plexin C1	10	BB476707
86	1460426_at	-1.0025	1.59E-07	7.65E-05	Pde4dip	phosphodiesterase 4D interacting protein (myomegalin)	3	AI639670
87	1432539_a_at	-1.00312	1.30E-05	0.000995262	Nup54	nucleoporin 54	5	AK014260
88	1453286_at	-1.00522	2.36E-06	0.000324077	Plxna2	plexin A2	1	BB085537
89	1449928_at	-1.00966	1.36E-07	6.98E-05	Dynlt3	dynein light chain Tctex-type 3	X	NM_025975
90	1421035_a_at	-1.0108	5.55E-08	5.56E-05	Magi3	membrane associated guanylate kinase, WW and PDZ domain containing 3	3	BB329638
91	1422474_at	-1.01265	0.00092442	0.0157485	Pde4b	phosphodiesterase 4B, cAMP specific	4	BM246564
92	1417831_at	-1.01526	4.52E-07	0.000121643	Smc1a	structural maintenance of chromosomes 1A	X	BB156359
93	1438403_s_at	-1.01803	0.0052635	0.0496559	Malat1	metastasis associated lung adenocarcinoma transcript 1 (non-coding RNA)	19	BF537798
94	1416403_at	-1.01959	2.19E-07	8.93E-05	Abcb10	ATP-binding cassette, sub-family B (MDR/TAP), member 10	8	AV382118
95	1416530_a_at	-1.02109	1.21E-07	6.57E-05				BC003788
96	1422528_a_at	-1.02178	0.000369504	0.00853881	Zfp36l1	zinc finger protein 36, C3H type-like 1	12	M58566
97	1436545_at	-1.02586	4.43E-06	0.00047586	Dtx4	deltex 4 homolog (Drosophila)	19	AV017487
98	1427319_at	-1.02616	4.32E-07	0.000119478	A230046K03Rik	RIKEN cDNA A230046K03 gene	10	AI607603
99	1427488_a_at	-1.02886	0.000187447	0.00543831	Birc6	baculoviral IAP repeat-containing 6	17	BC026990
100	1430053_a_at	-1.0298	1.21E-08	3.01E-05	Ola1	Obg-like ATPase 1	2	AK019142
101	1436746_at	-1.02995	9.87E-07	0.000189984	Wnk1	WNK lysine deficient protein kinase 1	6	BI692255
102	1427826_a_at	-1.03382	2.08E-05	0.00138023	Slco1b2	solute carrier organic anion transporter family, member 1b2	6	AB037192
103	1451018_at	-1.03427	1.21E-08	3.01E-05	Leprotl1	leptin receptor overlapping transcript-like 1	8	BF658789
104	1437901_a_at	-1.03431	1.79E-07	8.33E-05	Vps41	vacuolar protein sorting 41 (yeast)	13	BM240052
105	1455930_at	-1.03458	0.000142997	0.00453475				BI651113
106	1453259_at	-1.038	4.16E-05	0.0020897	Insc	inscuteable homolog (Drosophila)	7	BB667513
107	1431056_a_at	-1.03933	8.25E-05	0.00320132	Lpl	lipoprotein lipase	8	AK017272
108	1421230_a_at	-1.04054	2.90E-06	0.000361233	Msi2	Musashi homolog 2 (Drosophila)	11	BI696168
109	1422155_at	-1.04239	0.000437138	0.00949519	Hist2h3c2	histone cluster 2, H3c2	3	BC015270
110	1425206_a_at	-1.04279	1.09E-06	0.00020027	Ube3a	ubiquitin protein ligase E3A	7	BB224620
111	1456319_at	-1.04302	0.000290092	0.00724856				BG065719
112	1454633_at	-1.04495	1.63E-06	0.000255	Etnk1	ethanolamine kinase 1	6	BG066916
113	1418603_at	-1.04588	0.00145835	0.0211025	Avpr1a	arginine vasopressin receptor 1A	10	D49729
114	1424709_at	-1.04649	3.46E-06	0.000405096	Sc5d	sterol-C5-desaturase (fungal ERG3, delta-5-desaturase) homolog (S. cerevisae)	9	AB016248
115	1417028_a_at	-1.0587	9.57E-09	3.01E-05	Trim2	tripartite motif-containing 2	3	BB283676
116	1418227_at	-1.05992	4.83E-07	0.000126367	Orc2l	origin recognition complex, subunit 2-like (S. cerevisiae)	1	BB830976
117	1456120_at	-1.06041	2.12E-07	8.93E-05	Secisbp2l	SECIS binding protein 2-like	2	BB427489
118	1415988_at	-1.06177	7.85E-05	0.00311707	Hdlbp	high density lipoprotein (HDL) binding protein	1	BG065877
119	1431098_at	-1.06491	2.22E-05	0.00143469	Clip1	CAP-GLY domain containing linker protein 1	5	AK014540

120	1418660_at	-1.07041	3.56E-08	4.35E-05	Clock	circadian locomoter output cycles kaput	5	BB203106
121	1427430_at	-1.07095	7.76E-05	0.00309929	AI848100	expressed sequence AI848100	1	BB148987
122	1436931_at	-1.07621	0.0033143	0.0370246	Rfx4	regulatory factor X, 4 (influences HLA class II expression)	10	AV255458
123	1453414_at	-1.07795	0.000126051	0.00416879	Ypel2	yippee-like 2 (Drosophila)	11	BB133023
124	1434644_at	-1.07848	3.47E-05	0.0018842	Tbl1x	transducin (beta)-like 1 X-linked	X	BF682509
125	1434282_at	-1.08226	4.94E-06	0.000511639	Ibtk	inhibitor of Bruton agammaglobulinemia tyrosine kinase	9	BM250711
126	1417623_at	-1.08336	0.000215781	0.00592579	Slc12a2	solute carrier family 12, member 2	18	BG069505
127	1449578_at	-1.08514	0.00509463	0.0485823	Supt16h	suppressor of Ty 16 homolog (S. cerevisiae)	14	AW536705
128	1422741_a_at	-1.08693	1.64E-05	0.00115734	Bbx	bobby sox homolog (Drosophila)	16	BF319769
129	1455293_at	-1.08899	3.21E-07	0.000106211	Leo1	Leo1, Paf1/RNA polymerase II complex component, homolog (S. cerevisiae)	9	BG065311
130	1456088_at	-1.09327	1.85E-06	0.000276462	Xiap	X-linked inhibitor of apoptosis	X	BF134200
131	1431212_a_at	-1.09731	9.79E-08	5.74E-05	Trmt6	tRNA methyltransferase 6 homolog (S. cerevisiae)	2	BG079674
132	1453684_s_at	-1.09753	9.95E-07	0.000189984	Zc3h15	zinc finger CCCH-type containing 15	2	AK005661
133	1423571_at	-1.10491	2.78E-06	0.000353669	S1pr1	sphingosine-1-phosphate receptor 1	3	BB133079
134	1442939_at	-1.11173	4.05E-06	0.000447528	Rif1	Rap1 interacting factor 1 homolog (yeast)	2	BG065807
135	1448183_a_at	-1.11236	1.73E-06	0.000265269	Hif1a	hypoxia inducible factor 1, alpha subunit	12	BB269715
136	1424768_at	-1.11544	1.19E-07	6.57E-05	Cald1	caldesmon 1	6	BI248947
137	1426636_a_at	-1.11717	3.17E-06	0.000379801	Xiap	X-linked inhibitor of apoptosis	X	BB783769
138	1420941_at	-1.11884	4.65E-05	0.0022263	Rgs5	regulator of G-protein signaling 5	1	BF585144
139	1438804_at	-1.12113	3.70E-05	0.00195938	9-Sep	septin 10	10	AV254985
140	1416732_at	-1.12259	1.16E-07	6.52E-05	Top2b	topoisomerase (DNA) II beta	14	BB166592
141	1423184_at	-1.12491	1.13E-07	6.51E-05	Itsn2	intersectin 2	12	AI326108
142	1450051_at	-1.12984	1.47E-06	0.000236443	Atrx	alpha thalassemia/mental retardation syndrome X-linked homolog (human)	X	BB825830
143	1428547_at	-1.13029	2.81E-06	0.000356514	Nt5e	5' nucleotidase, ecto	9	AV273591
144	1443889_at	-1.13196	0.00252784	0.0305699	9030619P08Rik	RIKEN cDNA 9030619P08 gene	15	AI789751
145	1418659_at	-1.13267	5.62E-06	0.000558514	Clock	circadian locomoter output cycles kaput	5	BB203106
146	1456767_at	-1.13909	9.75E-05	0.00358805	Lrfn3	leucine rich repeat and fibronectin type III domain containing 3	7	AV062156
147	1449931_at	-1.14329	8.71E-06	0.000753023	Cpeb4	cytoplasmic polyadenylation element binding protein 4	11	NM_026252
148	1418188_a_at	-1.15527	0.00245426	0.030027				AF146523
149	1429772_at	-1.16055	9.07E-08	5.70E-05	Plxna2	plexin A2	1	BB085537
150	1425742_a_at	-1.16247	0.000779771	0.0140955	Tsc22d1	TSC22 domain family, member 1	14	AF201285
151	1416421_a_at	-1.16429	6.67E-08	5.70E-05	Ssb	Sjogren syndrome antigen B	2	BG796845
152	1460729_at	-1.16434	2.39E-06	0.000325508	Rock1	Rho-associated coiled-coil containing protein kinase 1	18	BI662863
153	1420479_a_at	-1.16858	8.14E-07	0.000177992	Nap1l1	nucleosome assembly protein 1-like 1	10	BG064031
154	1458539_at	-1.18178	2.85E-07	9.93E-05	R3hdm1	R3H domain 1 (binds single-stranded nucleic acids)	1	BB462088

155	1427037_at	-1.18376	1.09E-05	0.000877944	Eif4g1	eukaryotic translation initiation factor 4, gamma 1	16	BF227830
156	1421885_at	-1.18541	4.90E-10	1.28E-05	Sos1	son of sevenless homolog 1 (Drosophila)	17	BB471450
157	1436181_at	-1.18647	7.17E-06	0.000652303	Asap2	ArfGAP with SH3 domain, ankyrin repeat and PH domain 2	12	AV077160
158	1452661_at	-1.18719	0.036628	0.17322	Tfrc	transferrin receptor	16	AK011596
159	1456827_at	-1.18755	7.73E-06	0.000690167				BB131790
160	1438264_a_at	-1.18756	9.62E-07	0.000189984	Tpp2	tripeptidyl peptidase II	1	AW536258
161	1437073_x_at	-1.19284	0.00228917	0.0285402				BB115446
162	1433453_a_at	-1.19616	0.000686104	0.0129454	Abtb2	ankyrin repeat and BTB (POZ) domain containing 2	2	BB621938
163	1438713_at	-1.1977	4.28E-07	0.00011939	Rassf8	Ras association (RalGDS/AF-6) domain family (N-terminal) member 8	6	BB391868
164	1450010_at	-1.19803	1.95E-08	3.18E-05	Hsd17b12	hydroxysteroid (17-beta) dehydrogenase 12	2	AK012103
165	1444512_at	-1.20004	0.000132298	0.00430734	Arhgap29	Rho GTPase activating protein 29	3	AI643890
166	1419497_at	-1.2051	1.61E-06	0.000254163	Cdkn1b	cyclin-dependent kinase inhibitor 1B	6	NM_009875
167	1459860_x_at	-1.20642	2.31E-08	3.56E-05	Trim2	tripartite motif-containing 2	3	BB466780
168	1452378_at	-1.21461	0.00210728	0.0269513	Malat1	metastasis associated lung adenocarcinoma transcript 1 (non-coding RNA)	19	AW012617
169	1448551_a_at	-1.22642	5.01E-07	0.000129948	Trim2	tripartite motif-containing 2	3	BB283676
170	1424318_at	-1.22749	0.000272504	0.00693737	1110067D22Rik	RIKEN cDNA 1110067D22 gene	11	BC019131
171	1449514_at	-1.2283	8.21E-08	5.70E-05	Grk5	G protein-coupled receptor kinase 5	19	BC019379
172	1428372_at	-1.22891	1.50E-06	0.000239345	St5	suppression of tumorigenicity 5	7	AK008100
173	1439158_at	-1.23171	1.48E-06	0.000237306	Tlk1	tousled-like kinase 1	2	BB749708
174	1427408_a_at	-1.23521	1.94E-05	0.00131035	Thrap3	thyroid hormone receptor associated protein 3	4	BC012655
175	1437609_at	-1.2415	9.08E-06	0.000775618	Ube2u	ubiquitin-conjugating enzyme E2U (putative)	4	AV278535
176	1417496_at	-1.24337	2.33E-07	8.93E-05	Cp	ceruloplasmin	3	BB332449
177	1429533_at	-1.25295	1.02E-06	0.000189984	Immt	inner membrane protein, mitochondrial	6	BB222675
178	1457554_at	-1.25642	3.37E-05	0.00185211	Apob	apolipoprotein B	12	BM251092
179	1424029_at	-1.26856	0.000459005	0.0098461	Tspyl4	TSPY-like 4	10	BC017540
180	1424486_a_at	-1.27129	2.88E-07	9.93E-05	Txnrd1	thioredoxin reductase 1	10	BB284199
181	1442367_at	-1.28575	4.25E-06	0.000462908	Atp11c	ATPase, class VI, type 11C	X	BB184010
182	1450090_at	-1.28734	9.53E-07	0.000189962	Zfp101	zinc finger protein 101	17	NM_009542
183	1450018_s_at	-1.29313	0.0031492	0.0358639	Slc25a30	solute carrier family 25, member 30	14	BB032012
184	1438269_at	-1.295	4.39E-07	0.00012023	Zbtb38	zinc finger and BTB domain containing 38	9	BB278987
185	1451313_a_at	-1.29648	0.00108866	0.0175371	1110067D22Rik	RIKEN cDNA 1110067D22 gene	11	BC019131
186	1433446_at	-1.29922	3.80E-05	0.00198593	Hmgcs1	3-hydroxy-3-methylglutaryl-Coenzyme A synthase 1	13	BB705380
187	1450846_at	-1.3017	7.24E-06	0.000656946	Bzw1	basic leucine zipper and W2 domains 1	1	AV144956
188	1423397_at	-1.30314	0.00159809	0.0223839				AI118428
189	1443901_at	-1.30719	1.50E-07	7.31E-05	C2cd2	C2 calcium-dependent domain containing 2	16	BB481579
190	1452030_a_at	-1.30993	8.26E-08	5.70E-05	Hnrnpr	heterogeneous nuclear ribonucleoprotein R	4	BB822465
191	1433515_s_at	-1.31147	1.44E-08	3.01E-05	Etnk1	ethanolamine kinase 1	6	BG066916
192	1420917_at	-1.31895	8.41E-07	0.000179812	Prpf40a	PRP40 pre-mRNA processing factor 40 homolog A (yeast)	2	BG064340

193	1435775_at	-1.31952	8.62E-08	5.70E-05	Clock	circadian locomoter output cycles kaput	5	BQ173970
194	1427574_s_at	-1.32668	1.71E-06	0.000264709	Sh3d19	SH3 domain protein D19	3	BF232848
195	1417832_at	-1.3413	3.13E-09	1.84E-05	Smc1a	structural maintenance of chromosomes 1A	X	BB156359
196	1450035_a_at	-1.35058	3.02E-07	0.00010223	Prpf40a	PRP40 pre-mRNA processing factor 40 homolog A (yeast)	2	BG064340
197	1437932_a_at	-1.35605	6.57E-05	0.00279332	Cldn1	claudin 1	16	AV227581
198	1433898_at	-1.36753	0.00283465	0.0332979				AV000840
199	1457758_at	-1.3842	2.87E-09	1.84E-05	Eny2	enhancer of yellow 2 homolog (Drosophila)	15	BB055459
200	1423447_at	-1.40514	8.33E-06	0.000733154	Clpx	caseinolytic peptidase X (E.coli)	9	BF020441
201	1450743_s_at	-1.4165	3.35E-07	0.00010772	Syncrip	synaptotagmin binding, cytoplasmic RNA interacting protein	9	BG920261
202	1431024_a_at	-1.41823	2.81E-05	0.00165918	Arid4b	AT rich interactive domain 4B (RBP1-like)	13	AK020165
203	1440522_at	-1.42675	9.15E-08	5.70E-05	Gm10454	predicted gene 10454	X	BB349472
204	1438751_at	-1.45438	4.47E-05	0.00218559	Slc30a10	solute carrier family 30, member 10	1	BB736474
205	1452445_at	-1.45681	0.000155482	0.00475889	Slc41a2	solute carrier family 41, member 2	10	BC026874
206	1425099_a_at	-1.47001	0.000413267	0.00916261	Arntl	aryl hydrocarbon receptor nuclear translocator-like	7	BC011080
207	1433944_at	-1.4783	9.70E-07	0.000189984	Hectd2	HECT domain containing 2	19	AV256030
208	1450264_a_at	-1.48087	3.01E-05	0.0017271	Chka	choline kinase alpha	19	NM_013490
209	1423325_at	-1.49602	5.89E-06	0.000573529	Pnn	pinin	12	AV135835
210	1426458_at	-1.52054	2.31E-07	8.93E-05	Slmap	sarcolemma associated protein	14	BB473571
211	1437581_at	-1.53472	3.91E-06	0.00043727	Zfp800	zinc finger protein 800	6	AW824355
212	1426645_at	-1.54547	8.41E-05	0.00325938	Hsp90aa1	heat shock protein 90, alpha (cytosolic), class A member 1	12	AU079047
213	1417980_a_at	-1.55575	2.41E-06	0.000327028	Insig2	insulin induced gene 2	1	AV257512
214	1422769_at	-1.57914	1.40E-06	0.000232284	Syncrip	synaptotagmin binding, cytoplasmic RNA interacting protein	9	BG920261
215	1417981_at	-1.58882	6.70E-07	0.00016089	Insig2	insulin induced gene 2	1	AV257512
216	1417982_at	-1.81288	4.47E-08	5.04E-05	Insig2	insulin induced gene 2	1	AV257512
217	1421447_at	-1.83832	0.0236092	0.131838				NM_008262
218	1427838_at	-1.84653	2.44E-05	0.00153458	Tubb2a	tubulin, beta 2A	13	M28739
219	1420722_at	-1.97947	2.22E-06	0.000312665	Elovl3	elongation of very long chain fatty acids (FEN1/Elo2, SUR4/Elo3, yeast)-like 3	19	BC016468
220	1427347_s_at	-2.06277	7.86E-05	0.003118	Tubb2a	tubulin, beta 2A	13	BC003475
221	1426215_at	-2.10055	1.08E-08	3.01E-05	Ddc	dopa decarboxylase	11	AF071068
222	1439300_at	-2.19417	3.08E-08	4.10E-05	Chic1	cysteine-rich hydrophobic domain 1	X	BG065782

SHS^{4} & SHS^{4+1R} vs C

	Probeset_id	Log2Ratio.1	P.Value	Adj.P.Value	Symbol	Description	Chromosome	GenBank
1	1426037_a_at	3.54318	2.81E-06	0.000527558	Rgs16	regulator of G-protein signaling 16	1	U94828
2	1422557_s_at	3.46629	0.00403622	0.0410515	Mt1	metallothionein 1	8	NM_013602
3	1428942_at	3.26031	0.0206807	0.115053	Mt2	metallothionein 2	8	AA796766
4	1417168_a_at	3.24514	1.70E-09	1.30E-05	Usp2	ubiquitin specific peptidase 2	9	AI553394

5	1422257_s_at	2.94269	0.00184722	0.0246219	Cyp2b10	cytochrome P450, family 2, subfamily b, polypeptide 10	7	NM_009998
6	1425645_s_at	2.88808	0.00196963	0.0257079	Cyp2b10	cytochrome P450, family 2, subfamily b, polypeptide 10	7	AF128849
7	1417169_at	2.86908	2.58E-10	7.57E-06	Usp2	ubiquitin specific peptidase 2	9	AI553394
8	1455265_a_at	2.86352	1.14E-06	0.00031903	Rgs16	regulator of G-protein signaling 16	1	BB100249
9	1451787_at	2.61325	0.00214069	0.0272347	Cyp2b10	cytochrome P450, family 2, subfamily b, polypeptide 10	7	AF128849
10	1442025_a_at	2.38743	0.0014187	0.0205469				AI467657
11	1419874_x_at	2.28091	0.000836699	0.0145848	Zbtb16	zinc finger and BTB domain containing 16	9	AA419994
12	1418288_at	2.26513	1.04E-05	0.00103098	Lpin1	lipin 1	12	NM_015763
13	1427747_a_at	2.22071	0.0325637	0.152134	Lcn2	lipocalin 2	2	X14607
14	1428223_at	2.17402	1.37E-05	0.00120468	Mfsd2a	major facilitator superfamily domain containing 2A	4	AK006096
15	1443147_at	2.16824	0.0215029	0.118058				BB505010
16	1426516_a_at	2.15556	1.58E-05	0.00128344	Lpin1	lipin 1	12	AK014526
17	1416125_at	2.09521	1.15E-05	0.00108669	Fkbp5	FK506 binding protein 5	17	U16959
18	1435188_at	1.95613	9.20E-08	8.43E-05	Gm129	predicted gene 129	3	BB407125
19	1425837_a_at	1.93271	0.000598088	0.0118333				AF199491
20	1451190_a_at	1.92231	7.43E-08	7.26E-05	Sbk1	SH3-binding kinase 1	7	BC025837
21	1428923_at	1.87038	0.000149146	0.00492702	Ppp1r3g	protein phosphatase 1, regulatory (inhibitor) subunit 3G	13	AK005570
22	1451548_at	1.86397	6.81E-06	0.000826531	Upp2	uridine phosphorylase 2	2	BC027189
23	1422925_s_at	1.84538	0.00629946	0.0545645	Acot3	acyl-CoA thioesterase 3	12	NM_134246
24	1424969_s_at	1.80657	2.79E-06	0.000527558	Upp2	uridine phosphorylase 2	2	BC027189
25	1460241_a_at	1.77415	0.00023503	0.0066156	St3gal5	ST3 beta-galactoside alpha-2,3-sialyltransferase 5	6	BB829192
26	1419590_at	1.75875	0.00847351	0.0656702				NM_010000
27	1416432_at	1.70676	0.000179236	0.00556353	Pfkfb3	6-phosphofructo-2-kinase/fructose-2,6-biphosphatase 3	2	NM_133232
28	1423978_at	1.70474	4.55E-08	6.08E-05	Sbk1	SH3-binding kinase 1	7	BC025837
29	1442026_at	1.6864	0.00176898	0.0239321				AI467657
30	1429144_at	1.60963	0.000283128	0.00749433	Gpcpd1	glycerophosphocholine phosphodiesterase GDE1 homolog (S. cerevisiae)	2	AV291259
31	1434437_x_at	1.59806	9.73E-05	0.00381855	Rrm2	ribonucleotide reductase M2	12	AV301324
32	1416933_at	1.59443	1.60E-07	0.000118507	Por	P450 (cytochrome) oxidoreductase	5	NM_008898
33	1449198_a_at	1.57747	3.08E-05	0.00190673	St3gal5	ST3 beta-galactoside alpha-2,3-sialyltransferase 5	6	BB829192
34	1439489_at	1.54199	0.0150026	0.094154	Gpr120	G protein-coupled receptor 120	19	AV025152
35	1448162_at	1.54189	2.88E-06	0.000534906	Vcam1	vascular cell adhesion molecule 1	3	BB250384
36	1451452_a_at	1.53792	1.05E-06	0.000314071	Rgs16	regulator of G-protein signaling 16	1	U72881
37	1429206_at	1.52315	2.01E-07	0.000123167	Rhobtb1	Rho-related BTB domain containing 1	10	AK014194
38	1443137_at	1.52178	0.0159406	0.0978818				BB534298
39	1453023_at	1.51806	0.000190435	0.00580111				AK003441
40	1434473_at	1.49278	0.00189028	0.0250191	Slc16a5	solute carrier family 16 (monocarboxylic acid transporters), member 5	11	AI647939
41	1417761_at	1.49219	0.00402874	0.0410426	Apoa4	apolipoprotein A-IV	9	BC010769

42	1439163_at	1.48265	0.00682332	0.0573542	Zbtb16	zinc finger and BTB domain containing 16	9	BQ174973
43	1417602_at	1.46931	4.50E-07	0.000188234	Per2	period homolog 2 (Drosophila)	1	AF035830
44	1425824_a_at	1.44956	1.63E-05	0.00130796	Pcsk4	proprotein convertase subtilisin/kexin type 4	10	D01093
45	1448239_at	1.43744	0.00090093	0.015285	Hmox1	heme oxygenase (decycling) 1	8	NM_010442
46	1452416_at	1.43303	1.14E-06	0.00031903	Il6ra	interleukin 6 receptor, alpha	3	X53802
47	1427473_at	1.41786	0.0335956	0.155242	Gstm3	glutathione S-transferase, mu 3	3	J03953
48	1460059_at	1.41553	4.05E-05	0.0022902	Upp2	uridine phosphorylase 2	2	BB272732
49	1449851_at	1.40328	1.38E-06	0.000344837	Per1	period homolog 1 (Drosophila)	11	AF022992
50	1452426_x_at	1.38167	0.00134302	0.0198246				BC004065
51	1417904_at	1.38009	0.000459866	0.0100983	Dclre1a	DNA cross-link repair 1A, PSO2 homolog (S. cerevisiae)	19	AF241240
52	1456960_at	1.34477	0.0222761	0.120742				BB555069
53	1448226_at	1.33683	0.00117333	0.0181621	Rrm2	ribonucleotide reductase M2	12	NM_009104
54	1436504_x_at	1.33662	0.00545864	0.0497344	Apoa4	apolipoprotein A-IV	9	AV027367
55	1450505_a_at	1.33564	0.00100863	0.0164284	Fam134b	family with sequence similarity 134, member B	15	NM_025459
56	1440840_at	1.33428	7.62E-09	2.22E-05	D630004K10Rik	RIKEN cDNA D630004K10 gene	10	BB335455
57	1423233_at	1.3023	0.000901649	0.015285	Cebpd	CCAAT/enhancer binding protein (C/EBP), delta	16	BB831146
58	1416773_at	1.29636	3.82E-06	0.000625046	Wee1	WEE 1 homolog 1 (S. pombe)	7	NM_009516
59	1460510_a_at	1.28756	2.23E-06	0.000448303	Coq10b	coenzyme Q10 homolog B (S. cerevisiae)	1	AK006551
60	1435459_at	1.27914	0.000295712	0.00770217	Fmo2	flavin containing monooxygenase 2	1	BM936480
61	1426452_a_at	1.2788	0.0281134	0.139011	Rab30	RAB30, member RAS oncogene family	7	BG070713
62	1458040_at	1.272	0.000523413	0.0108197				BM213832
63	1421681_at	1.2682	8.48E-05	0.00350633	Nrg4	neuregulin 4	9	NM_032002
64	1437953_at	1.26172	0.000148845	0.00492262	Gpcpd1	glycerophosphocholine phosphodiesterase GDE1 homolog (S. cerevisiae)	2	BM246706
65	1435495_at	1.25937	0.00156021	0.0219373	Adora1	adenosine A1 receptor	1	BE630294
66	1426850_a_at	1.25071	7.94E-05	0.00333718	Map2k6	mitogen-activated protein kinase kinase 6	11	BB261602
67	1421852_at	1.24705	0.00102077	0.0165802	Kcnk5	potassium channel, subfamily K, member 5	14	AF319542
68	1443870_at	1.24644	0.000129742	0.00456691	Abcc4	ATP-binding cassette, sub-family C (CFTR/MRP), member 4	14	BB291885
69	1441971_at	1.24317	0.000586489	0.0116802				AW543723
70	1422230_s_at	1.24154	2.41E-08	4.70E-05				NM_007812
71	1458442_at	1.23946	0.00785727	0.0625635	AI132709	expressed sequence AI132709	7	AI266897
72	1440084_at	1.23942	0.00144152	0.0208178				AV380966
73	1424175_at	1.23833	5.62E-08	6.58E-05	Tef	thyrotroph embryonic factor	15	BC017689
74	1424744_at	1.2307	0.00292498	0.0336637	Sds	serine dehydratase	5	BC021950
75	1434292_at	1.21291	0.000850327	0.0147608	Snhg11	small nucleolar RNA host gene 11 (non-protein coding)	2	BI731047
76	1418780_at	1.21152	0.0031624	0.0353412	Cyp39a1	cytochrome P450, family 39, subfamily a, polypeptide 1	17	NM_018887
77	1453410_at	1.20724	0.0112369	0.0785831	Angptl4	angiopoietin-like 4	17	AK014564
78	1456156_at	1.19909	6.39E-05	0.00295409	Lepr	leptin receptor	4	BM124366
79	1449498_at	1.19846	0.000447822	0.00994096	Marco	macrophage receptor with collagenous structure	1	NM_010766
80	1449565_at	1.19834	6.89E-06	0.000826531	Cyp2g1	cytochrome P450, family 2, subfamily g, polypeptide 1	7	NM_013809

81	1428352_at	1.19705	0.00305943	0.0346129	Arrdc2	arrestin domain containing 2	8	AW542672
82	1418595_at	1.18548	0.000919408	0.0154742	Plin4	perilipin 4	17	NM_020568
83	1417042_at	1.17816	4.03E-07	0.000176051	Slc37a4	solute carrier family 37 (glucose-6-phosphate transporter), member 4	9	NM_008063
84	1451612_at	1.17116	0.0136109	0.0885955	Mt1	metallothionein 1	8	BC027262
85	1426980_s_at	1.16895	0.000329457	0.00825757	E130012A19Rik	RIKEN cDNA E130012A19 gene	11	BC006054
86	1445574_at	1.16415	0.000130946	0.00457327				BG067678
87	1429809_at	1.16207	2.22E-09	1.30E-05	Tmtc2	transmembrane and tetratricopeptide repeat containing 2	10	AK018506
88	1455958_s_at	1.15244	0.00183218	0.0244781	Pptc7	PTC7 protein phosphatase homolog (S. cerevisiae)	5	AI881989
89	1431339_a_at	1.15051	4.15E-05	0.00231736	Efhd2	EF hand domain containing 2	4	AK007560
90	1428512_at	1.14241	0.00647051	0.0555029	Bhlhb9	basic helix-loop-helix domain containing, class B9	X	AK012577
91	1455002_at	1.14129	3.19E-06	0.000566639	Ptp4a1	protein tyrosine phosphatase 4a1	1	AV331223
92	1428926_at	1.13272	2.62E-05	0.00170473	1110003O08Rik	RIKEN cDNA 1110003O08 gene	8	AK003388
93	1457123_at	1.1248	0.00112945	0.0177168	Nrg4	neuregulin 4	9	BB219343
94	1416286_at	1.12282	0.00789314	0.0627638	Rgs4	regulator of G-protein signaling 4	1	NM_009062
95	1432543_a_at	1.1154	1.98E-06	0.000420999	Klf13	Kruppel-like factor 13	7	AK002926
96	1434456_at	1.11091	1.00E-05	0.00101727	Rundc3b	RUN domain containing 3B	5	BG075955
97	1435860_at	1.1079	6.79E-08	7.18E-05	Slc5a6	solute carrier family 5 (sodium-dependent vitamin transporter), member 6	5	BF450030
98	1427912_at	1.10241	0.0152692	0.0949707	Cbr3	carbonyl reductase 3	16	AK003232
99	1456395_at	1.09807	0.0034357	0.0371761	Ppargc1a	peroxisome proliferative activated receptor, gamma, coactivator 1 alpha	5	BM120569
100	1419857_at	1.09565	0.0316911	0.149247				AA254866
101	1454799_at	1.09081	0.00389501	0.0402581	Agpat9	1-acylglycerol-3-phosphate O-acyltransferase 9	5	AV300264
102	1449945_at	1.06025	3.10E-05	0.00191379	Ppargc1b	peroxisome proliferative activated receptor, gamma, coactivator 1 beta	18	NM_133249
103	1434099_at	1.04721	0.00151229	0.0215426	Ppargc1a	peroxisome proliferative activated receptor, gamma, coactivator 1 alpha	5	BB752393
104	1419758_at	1.04695	0.000727962	0.0133735	Abcb1a	ATP-binding cassette, sub-family B (MDR/TAP), member 1A	5	M30697
105	1422905_s_at	1.04498	0.000768908	0.0138309	Fmo2	flavin containing monooxygenase 2	1	NM_018881
106	1437478_s_at	1.04052	3.11E-06	0.000555278	Efhd2	EF hand domain containing 2	4	AA409309
107	1423627_at	1.03984	0.00234188	0.0288322	Nqo1	NAD(P)H dehydrogenase, quinone 1	8	AV158882
108	1438211_s_at	1.0364	0.0498512	0.199174	Dbp	D site albumin promoter binding protein	7	BB550183
109	1424815_at	1.03202	2.25E-05	0.00157535	Gys2	glycogen synthase 2	6	BC021322
110	1424683_at	1.0287	0.00296526	0.0339938	Fam134b	family with sequence similarity 134, member B	15	BC019494
111	1419024_at	1.02753	0.000709174	0.0131938	Ptp4a1	protein tyrosine phosphatase 4a1	1	BC003761
112	1434100_x_at	1.02553	0.00123889	0.0188093	Ppargc1a	peroxisome proliferative activated receptor, gamma, coactivator 1 alpha	5	BB752393
113	1417969_at	1.02292	1.83E-05	0.00138468	Fbxo31	F-box protein 31	8	NM_133765
114	1448568_a_at	1.02106	1.99E-05	0.00145969	Slc20a1	solute carrier family 20, member 1	2	NM_015747
115	1420772_a_at	1.00747	0.00907088	0.0684157	Tsc22d3	TSC22 domain family, member 3	X	NM_010286

116	1428487_s_at	1.00705	0.00109365	0.017388	Coq10b	coenzyme Q10 homolog B (S. cerevisiae)	1	AK002294
117	1433816_at	1.00556	0.000868251	0.0149832	Mcart1	mitochondrial carrier triple repeat 1	4	BQ031264
118	1437751_at	1.00301	0.0151233	0.0946258	Ppargc1a	peroxisome proliferative activated receptor, gamma, coactivator 1 alpha	5	AV337619
119	1439377_x_at	-1.00348	0.00165719	0.0228728	Cdc20	cell division cycle 20 homolog (S. cerevisiae)	4	BB041150
120	1455293_at	-1.00569	7.16E-08	7.23E-05	Leo1	Leo1, Paf1/RNA polymerase II complex component, homolog (S. cerevisiae)	9	BG065311
121	1431056_a_at	-1.00785	1.27E-05	0.00116774	Lpl	lipoprotein lipase	8	AK017272
122	1450010_at	-1.01328	2.10E-07	0.000123167	Hsd17b12	hydroxysteroid (17-beta) dehydrogenase 12	2	AK012103
123	1433444_at	-1.01491	0.000829522	0.0145245	Hmgcs1	3-hydroxy-3-methylglutaryl-Coenzyme A synthase 1	13	BB705380
124	1454633_at	-1.01541	1.26E-07	0.000105868	Etnk1	ethanolamine kinase 1	6	BG066916
125	1417792_at	-1.0218	3.92E-07	0.000174198	Zfml	zinc finger, matrin-like	6	BM238431
126	1417292_at	-1.02183	6.83E-06	0.000826531	Ifi47	interferon gamma inducible protein 47	11	NM_008330
127	1452445_at	-1.0271	0.00345723	0.0373264	Slc41a2	solute carrier family 41, member 2	10	BC026874
128	1433443_a_at	-1.0307	0.000751865	0.01365	Hmgcs1	3-hydroxy-3-methylglutaryl-Coenzyme A synthase 1	13	BB705380
129	1423804_a_at	-1.03079	0.0319405	0.149987	Idi1	isopentenyl-diphosphate delta isomerase	13	BC004801
130	1420917_at	-1.03456	6.36E-05	0.00295261	Prpf40a	PRP40 pre-mRNA processing factor 40 homolog A (yeast)	2	BG064340
131	1448986_x_at	-1.03579	0.036159	0.16297	Dnase2a	deoxyribonuclease II alpha	8	NM_010062
132	1436931_at	-1.03674	0.00897558	0.0680219	Rfx4	regulatory factor X, 4 (influences HLA class II expression)	10	AV255458
133	1450035_a_at	-1.03912	4.73E-05	0.00251328	Prpf40a	PRP40 pre-mRNA processing factor 40 homolog A (yeast)	2	BG064340
134	1428022_at	-1.04042	0.0311798	0.147932	Lcn13	lipocalin 13	2	BC027556
135	1427356_at	-1.0415	1.66E-05	0.00131889	Fam89a	family with sequence similarity 89, member A	8	BC023460
136	1424033_at	-1.0421	0.0046852	0.0451301	Sfrs7	splicing factor, arginine/serine-rich 7	17	BC014857
137	1457758_at	-1.04328	5.43E-06	0.000738943	Eny2	enhancer of yellow 2 homolog (Drosophila)	15	BB055459
138	1416403_at	-1.04537	2.70E-05	0.00173334	Abcb10	ATP-binding cassette, sub-family B (MDR/TAP), member 10	8	AV382118
139	1450846_at	-1.04658	5.25E-05	0.00266137	Bzw1	basic leucine zipper and W2 domains 1	1	AV144956
140	1435462_at	-1.04721	0.00813653	0.0640045	Plcxd2	phosphatidylinositol-specific phospholipase C, X domain containing 2	16	BQ176176
141	1438713_at	-1.04833	6.10E-07	0.000219561	Rassf8	Ras association (RalGDS/AF-6) domain family (N-terminal) member 8	6	BB391868
142	1433515_s_at	-1.05135	1.31E-06	0.00033455	Etnk1	ethanolamine kinase 1	6	BG066916
143	1437864_at	-1.05726	1.15E-06	0.00031903	Adipor2	adiponectin receptor 2	6	BE632137
144	1451122_at	-1.05744	0.0309792	0.147596	Idi1	isopentenyl-diphosphate delta isomerase	13	BC004801
145	1425206_a_at	-1.06114	4.34E-08	6.08E-05	Ube3a	ubiquitin protein ligase E3A	7	BB224620
146	1433445_x_at	-1.06127	0.000561088	0.0113386	Hmgcs1	3-hydroxy-3-methylglutaryl-Coenzyme A synthase 1	13	BB705380
147	1420379_at	-1.07315	0.0123942	0.0837219	Slco1a1	solute carrier organic anion transporter family, member 1a1	6	AB031813
148	1448183_a_at	-1.07652	7.05E-07	0.000232199	Hif1a	hypoxia inducible factor 1, alpha subunit	12	BB269715

149	1440522_at	-1.07682	0.000107488	0.00405878	Gm10454	predicted gene 10454	X	BB349472
150	1428372_at	-1.08492	1.79E-05	0.00135996	St5	suppression of tumorigenicity 5	7	AK008100
151	1452030_a_at	-1.08521	3.50E-06	0.000599552	Hnrnpr	heterogeneous nuclear ribonucleoprotein R	4	BB822465
152	1450484_a_at	-1.08572	4.17E-06	0.000649219	Cmpk2	cytidine monophosphate (UMP-CMP) kinase 2, mitochondrial	12	AK004595
153	1431024_a_at	-1.09081	0.000358396	0.00858686	Arid4b	AT rich interactive domain 4B (RBP1-like)	13	AK020165
154	1453286_at	-1.10227	4.94E-07	0.000195799	Plxna2	plexin A2	1	BB085537
155	1417832_at	-1.10807	1.96E-07	0.000123167	Smc1a	structural maintenance of chromosomes 1A	X	BB156359
156	1424842_a_at	-1.10854	5.84E-06	0.000774214	Arhgap24	Rho GTPase activating protein 24	5	BC025502
157	1455324_at	-1.10967	0.00356044	0.0380438	Plcxd2	phosphatidylinositol-specific phospholipase C, X domain containing 2	16	BQ176176
158	1426458_at	-1.11961	0.000115048	0.0042245	Slmap	sarcolemma associated protein	14	BB473571
159	1438676_at	-1.12775	0.0182889	0.106967	Mpa2l	macrophage activation 2 like	5	BM241485
160	1438269_at	-1.12831	6.46E-07	0.000225493	Zbtb38	zinc finger and BTB domain containing 38	9	BB278987
161	1449931_at	-1.1309	1.14E-06	0.00031903	Cpeb4	cytoplasmic polyadenylation element binding protein 4	11	NM_026252
162	1442537_at	-1.13626	0.00190417	0.0251093				BB771206
163	1449854_at	-1.1576	0.00602354	0.0531632	Nr0b2	nuclear receptor subfamily 0, group B, member 2	4	BC019540
164	1427574_s_at	-1.15973	9.38E-06	0.000984626	Sh3d19	SH3 domain protein D19	3	BF232848
165	1435775_at	-1.1648	5.13E-06	0.000718651	Clock	circadian locomoter output cycles kaput	5	BQ173970
166	1429772_at	-1.16933	6.10E-08	6.87E-05	Plxna2	plexin A2	1	BB085537
167	1437932_a_at	-1.17127	0.000187866	0.0057522	Cldn1	claudin 1	16	AV227581
168	1423325_at	-1.17186	0.000102936	0.00395224	Pnn	pinin	12	AV135835
169	1447927_at	-1.1804	0.00921288	0.0691839	Mpa2l	macrophage activation 2 like	5	BG092512
170	1425099_a_at	-1.20471	0.00114204	0.0178815	Arntl	aryl hydrocarbon receptor nuclear translocator-like	7	BC011080
171	1444512_at	-1.20814	1.29E-05	0.00116875	Arhgap29	Rho GTPase activating protein 29	3	AI643890
172	1420835_at	-1.21438	0.000148556	0.00491863	Slc25a30	solute carrier family 25, member 30	14	BB032012
173	1442367_at	-1.23752	3.35E-06	0.000585788	Atp11c	ATPase, class VI, type 11C	X	BB184010
174	1450743_s_at	-1.25166	1.04E-06	0.00031344	Syncrip	synaptotagmin binding, cytoplasmic RNA interacting protein	9	BG920261
175	1417982_at	-1.26983	0.00495309	0.0467726	Insig2	insulin induced gene 2	1	AV257512
176	1427513_at	-1.27542	0.00118846	0.0183169	BC024137	cDNA sequence BC024137	8	BI144810
177	1430896_s_at	-1.27631	0.00193185	0.0253801	Nudt7	nudix (nucleoside diphosphate linked moiety X)-type motif 7	8	AK008824
178	1450090_at	-1.28931	8.71E-08	8.23E-05	Zfp101	zinc finger protein 101	17	NM_009542
179	1449514_at	-1.29532	9.08E-09	2.22E-05	Grk5	G protein-coupled receptor kinase 5	19	BC019379
180	1456074_at	-1.31597	0.000345726	0.00846981	Sdr9c7	4short chain dehydrogenase/reductase family 9C, member 7	10	BB143568
181	1421092_at	-1.32543	0.0147144	0.092943	Serpina12	serine (or cysteine) peptidase inhibitor, clade A (alpha-1 antiproteinase, antitrypsin), member 12	12	AK014346
182	1423571_at	-1.34272	7.65E-07	0.000243626	S1pr1	sphingosine-1-phosphate receptor 1	3	BB133079
183	1420531_at	-1.34599	0.0190975	0.109668	Hsd3b5	hydroxy-delta-5-steroid dehydrogenase, 3 beta- and steroid delta-isomerase 5	3	NM_008295

184	1422769_at		-1.35435	6.91E-06	0.000826531	Syncrip	synaptotagmin binding, cytoplasmic RNA interacting protein	9	BG920261
185	1437581_at		-1.37879	1.81E-06	0.000405871	Zfp800	zinc finger protein 800	6	AW824355
186	1430785_at		-1.39487	1.31E-05	0.00117124	Sdr9c7	4short chain dehydrogenase/reductase family 9C, member 7	10	BB150587
187	1426215_at		-1.40168	0.000522785	0.0108183	Ddc	dopa decarboxylase	11	AF071068
188	1426645_at		-1.40547	7.22E-05	0.00318597	Hsp90aa1	heat shock protein 90, alpha (cytosolic), class A member 1	12	AU079047
189	1433446_at		-1.43449	6.47E-06	0.000807214	Hmgcs1	3-hydroxy-3-methylglutaryl-Coenzyme A synthase 1	13	BB705380
190	1420836_at		-1.46472	0.000350378	0.00849491	Slc25a30	solute carrier family 25, member 30	14	BB032012
191	1434520_at		-1.46486	0.00219565	0.0276481	Sc5d	sterol-C5-desaturase (fungal ERG3, delta-5-desaturase) homolog (S. cerevisae)	9	AU067703
192	1423397_at		-1.47208	0.000188829	0.00577565				AI118428
193	1444296_a_at		-1.47292	0.0341278	0.156851	Serpina4-ps1	serine (or cysteine) peptidase inhibitor, clade A, member 4, pseudogene 1	12	BF383739
194	1424709_at		-1.48211	5.09E-06	0.000716971	Sc5d	sterol-C5-desaturase (fungal ERG3, delta-5-desaturase) homolog (S. cerevisae)	9	AB016248
195	1444297_at		-1.50419	0.0450803	0.186838	Serpina4-ps1	serine (or cysteine) peptidase inhibitor, clade A, member 4, pseudogene 1	12	BF383739
196	1450264_a_at		-1.50859	1.72E-06	0.000395031	Chka	choline kinase alpha	19	NM_013490
197	1438751_at		-1.55419	1.63E-06	0.000387662	Slc30a10	solute carrier family 30, member 10	1	BB736474
198	1427838_at		-1.57107	3.44E-05	0.00206133	Tubb2a	tubulin, beta 2A	13	M28739
199	1417065_at		-1.57312	0.00728926	0.0598132	Egr1	early growth response 1	18	NM_007913
200	1433944_at	-1.5796		2.29E-05	0.00158802	Hectd2	HECT domain containing 2	19	AV256030
201	1437073_x_at		-1.59842	0.00010628	0.00403101				BB115446
202	1431817_at		-1.62207	0.000564877	0.0113838	Adh6-ps1	alcohol dehydrogenase 6 (class V), pseudogene 1	3	AK004863
203	1439300_at		-1.66346	5.00E-05	0.00258327	Chic1	cysteine-rich hydrophobic domain 1	X	BG065782
204	1427347_s_at		-1.66595	0.000243917	0.0067875	Tubb2a	tubulin, beta 2A	13	BC003475
205	1450018_s_at		-1.69185	0.000133709	0.0046441	Slc25a30	solute carrier family 25, member 30	14	BB032012
206	1433898_at		-1.73693	0.000142778	0.00482756				AV000840
207	1450252_at		-1.80084	0.0117099	0.0807647	Onecut1	one cut domain, family member 1	9	NM_008262
208	1448092_x_at		-1.89626	0.0124239	0.0838234	Serpina4-ps1	serine (or cysteine) peptidase inhibitor, clade A, member 4, pseudogene 1	12	AA267743
209	1420722_at		-2.03359	6.86E-08	7.18E-05	Elovl3	elongation of very long chain fatty acids (FEN1/Elo2, SUR4/Elo3, yeast)-like 3	19	BC016468
210	1421447_at		-2.38181	0.00187298	0.0248786				NM_008262
